# Supplementary material for: Cross-sectional evaluation of exposure to ozone, nitrogen dioxide, and particulate mass levels on circulating immune markers in women in the California Teachers Study
Source: Sci Rep. 2025 Dec 5;16:1248. doi: 10.1038/s41598-025-30900-x (PMC12789436; doi:10.1038/s41598-025-30900-x)
Supplement: Supplementary file 1 — Supplementary Information. [file 41598_2025_30900_MOESM1_ESM.docx]

**Cross-sectional evaluation of exposure to ozone, nitrogen dioxide, and particulate mass levels on circulating immune markers in women in the California Teachers Study**

Supplemental Table S1. Spearman correlation values between the 15 serum-measured immune markers in 1,898 women in the California Teachers Study.

|  | **IL-1β** | **IL-6** | **IL-8** | **IL-10** | **TNFα** | **BAFF** | **CCL2** | **CCL17** | **sCD14** | **sCD25** | **sCD27** | **sCD163** | **sgp130** | **sIL-6Rα** | **sTNFR2** |
| --- | --- | --- | --- | --- | --- | --- | --- | --- | --- | --- | --- | --- | --- | --- | --- |
| **IL-1β** | 1.000 | 0.526 | 0.292 | 0.475 | 0.562 | 0.097 | 0.167 | -0.013 | -0.024 | 0.057 | 0.049 | 0.078 | 0.008 | 0.014 | 0.115 |
| **IL-6** |  | 1.000 | 0.129 | 0.566 | 0.660 | 0.195 | 0.213 | 0.022 | 0.115 | 0.160 | 0.149 | 0.203 | 0.065 | 0.124 | 0.212 |
| **IL-8** |  |  | 1.000 | 0.111 | 0.188 | 0.126 | 0.271 | 0.050 | 0.112 | 0.100 | 0.135 | 0.109 | 0.152 | 0.009 | 0.236 |
| **IL-10** |  |  |  | 1.000 | 0.627 | 0.173 | 0.163 | 0.055 | 0.049 | 0.145 | 0.130 | 0.116 | 0.044 | 0.045 | 0.159 |
| **TNFα** |  |  |  |  | 1.000 | 0.203 | 0.256 | 0.082 | 0.099 | 0.230 | 0.188 | 0.197 | 0.105 | 0.111 | 0.241 |
| **BAFF** |  |  |  |  |  | 1.000 | 0.282 | 0.170 | 0.259 | 0.381 | 0.263 | 0.306 | 0.246 | 0.180 | 0.377 |
| **CCL2** |  |  |  |  |  |  | 1.000 | 0.219 | 0.195 | 0.276 | 0.229 | 0.321 | 0.200 | 0.186 | 0.267 |
| **CCL17** |  |  |  |  |  |  |  | 1.000 | 0.080 | 0.239 | 0.236 | 0.222 | 0.176 | 0.216 | 0.197 |
| **sCD14** |  |  |  |  |  |  |  |  | 1.000 | 0.288 | 0.314 | 0.131 | 0.275 | 0.146 | 0.323 |
| **sCD25** |  |  |  |  |  |  |  |  |  | 1.000 | 0.568 | 0.387 | 0.215 | 0.291 | 0.616 |
| **sCD27** |  |  |  |  |  |  |  |  |  |  | 1.000 | 0.342 | 0.226 | 0.245 | 0.724 |
| **sCD163** |  |  |  |  |  |  |  |  |  |  |  | 1.000 | 0.307 | 0.261 | 0.458 |
| **sgp130** |  |  |  |  |  |  |  |  |  |  |  |  | 1.000 | 0.166 | 0.302 |
| **sIL-6Rα** |  |  |  |  |  |  |  |  |  |  |  |  |  | 1.000 | 0.266 |
| **sTNFR2** |  |  |  |  |  |  |  |  |  |  |  |  |  |  | 1.000 |

Supplemental Table S2. Pearson correlation values between the pollutants (measured in IQR) in 1,898 women in the California Teachers Study.

|  | Ozone (O_3_) | Nitrogen Dioxide (NO_2_) | PM_0.1_ Total Mass | PM_2.5_ Total Mass | PM_10_ Total Mass |
| --- | --- | --- | --- | --- | --- |
| Ozone (O_3_) | 1 | 0.33585 | 0.43674 | 0.28805 | 0.33319 |
| Nitrogen Dioxide (NO_2_) |  | 1 | 0.70696 | 0.85634 | 0.82707 |
| PM_0.1_ Total Mass |  |  | 1 | 0.74997 | 0.70624 |
| PM_2.5_ Total Mass |  |  |  | 1 | 0.93985 |
| PM_10_ Total Mass |  |  |  |  | 1 |

Supplemental Table S3. Comparison of study participant characteristics in the California Teachers Study cohort who had serum immune marker and pollutant measurements (N=1,898), participated in the larger biobanking study (N=13,888), and completed questionnaire 5 (N=61,984).

|  | | **Cohort with completed Q5 questionnaire (N=61,984 ^a^)** | | **Cohort with completed Q5 questionnaire and blood sample collected (N=13,888 ^a^)** | | **Cohort with cytokine and pollutant measurements (N=1,898^a^)** | | | |  |
| --- | --- | --- | --- | --- | --- | --- | --- | --- | --- | --- |
| **Characteristic** | | **N** | **%** | **N** | **%** | **N** | **%** |  |  |  |
| Age (in years)^b^ | |  |  |  |  |  |  |  |  |  |
|  | 40-49 | 4393 | 7.1% | 1333 | 9.6% | 206 | 10.9% |  |  |  |
|  | 50-59 | 10244 | 16.5% | 3452 | 24.9% | 551 | 29.0% |  |  |  |
|  | 60-69 | 22272 | 35.9% | 6586 | 47.4% | 795 | 41.9% |  |  |  |
|  | 70+ | 25044 | 40.4% | 2517 | 18.1% | 225 | 11.9% |  |  |  |
| Race^c^ | |  |  |  |  |  |  |  | | |
|  | Non-Hispanic White | 54294 | 87.6% | 12391 | 89.2% | 1456 | 76.7% |  |  |  |
|  | Other | 7690 | 12.4% | 1497 | 10.8% | 442 | 23.3% |  |  |  |
| Socioeconomic status (SES)^c^ | |  |  |  |  |  |  |  | | |
|  | Quartile 1 | 2376 | 3.9% | 457 | 3.3% | 163 | 8.6% |  |  |  |
|  | Quartile 2 | 9867 | 16.2% | 2155 | 15.7% | 725 | 38.2% |  |  |  |
|  | Quartile 3 | 19987 | 32.8% | 4607 | 33.5% | 449 | 23.7% |  |  |  |
|  | Quartile 4 | 28755 | 47.2% | 6533 | 47.5% | 544 | 28.7% |  |  |  |
| Body mass index (BMI)^b^ | |  |  |  |  |  |  |  | | |
|  | <25 | 29196 | 49.7% | 6744 | 50.1% | 804 | 42.4% |  |  |  |
|  | 25-29 | 17967 | 30.6% | 4056 | 30.1% | 536 | 28.2% |  |  |  |
|  | 30+ | 11580 | 19.7% | 2672 | 19.8% | 387 | 20.4% |  |  |  |
| Physical Activity (hr/week)^b^ | |  |  |  |  |  |  |  | | |
|  | 0-2.37 | 20947 | 34.3% | 3879 | 28.2% | 549 | 28.9% |  |  |  |
|  | 2.38-5.88 | 20136 | 33.0% | 4771 | 34.6% | 611 | 32.2% |  |  |  |
|  | 5.88+ | 20037 | 32.8% | 5132 | 37.2% | 605 | 31.9% |  |  |  |
| NSAID use^b^ | |  |  |  |  |  |  |  | | |
|  | None or 1/week | 24616 | 42.3% | 5784 | 43.3% | 771 | 40.6% |  |  |  |
|  | >1/week | 33630 | 57.7% | 7581 | 56.7% | 943 | 49.7% |  |  |  |
| Diabetes^b^ | |  |  |  |  |  |  |  | | |
|  | No | 56246 | 91.6% | 12891 | 93.4% | 1625 | 85.6% |  |  |  |
|  | Yes | 5128 | 8.4% | 907 | 6.6% | 142 | 7.5% |  |  |  |
| Statin use^b^ | |  |  |  |  |  |  |  | | |
|  | None | 42336 | 69.9% | 10188 | 74.4% | 1340 | 70.6% |  |  |  |
|  | >1/week | 18223 | 30.1% | 3513 | 25.6% | 417 | 22.0% |  |  |  |
| Rural/Urban residence^c^ | |  |  |  |  |  |  |  | | |
|  | Rural | 8813 | 14.4% | 1782 | 13.0% | 389 | 20.5% |  |  |  |
|  | Town | 2032 | 3.3% | 423 | 3.1% | 111 | 5.9% |  |  |  |
|  | City | 10905 | 17.9% | 2627 | 19.1% | 492 | 25.9% |  |  |  |
|  | Suburban | 33386 | 54.7% | 7586 | 55.1% | 732 | 38.6% |  |  |  |
|  | Urban | 5874 | 9.6% | 1342 | 9.8% | 159 | 8.4% |  |  |  |
| ^a^Sample sizes may not sum to the total sample size per group due to unknown covariate measures  ^b^Questionnaire 5 characteristic  ^c^Baseline characteristic (Questionnaire 1) | | | | | | | | |  |  |

Supplemental Table S4. Multivariable ordinal logistic regressions for the long-term (12-months) and short-term (3-months and 1-month) associations of ozone (O_3_) exposure with the 15 tested cytokines. Immune markers are reported as quartiles (reference is Quartile 1), and associations are scaled by IQR.

|  |  | **IL-1β** | | | | **IL-6** | | | | **sIL-6Rα** | | | | **IL-8** | | | | **IL-10** | | | |
| --- | --- | --- | --- | --- | --- | --- | --- | --- | --- | --- | --- | --- | --- | --- | --- | --- | --- | --- | --- | --- | --- |
|  | **Cytokine quartile** | **OR** | **L** | **U** | **P value** | **OR** | **L** | **U** | **P value** | **OR** | **L** | **U** | **P value** | **OR** | **L** | **U** | **P value** | **OR** | **L** | **U** | **P value** |
| 12-month | 2 | 1.05 | 0.82 | 1.34 | 0.7208 | 0.91 | 0.72 | 1.14 | 0.4017 | 0.97 | 0.78 | 1.21 | 0.7891 | 1.21 | 0.96 | 1.53 | 0.0993 | 1.12 | 0.86 | 1.45 | 0.4058 |
|  | 3 | 1.28 | 1.00 | 1.63 | 0.0529* | 1.22 | 0.97 | 1.53 | 0.0956 | 1.00 | 0.80 | 1.24 | 0.9625 | 1.16 | 0.92 | 1.46 | 0.2228 | 1.06 | 0.81 | 1.37 | 0.6835 |
|  | 4 | 1.99 | 1.55 | 2.56 | <.0001* | 1.07 | 0.85 | 1.35 | 0.5686 | 0.98 | 0.78 | 1.23 | 0.8643 | 2.92 | 2.30 | 3.71 | <.0001* | 1.08 | 0.83 | 1.40 | 0.5569 |
| 3-month | 2 | 0.99 | 0.77 | 1.26 | 0.9052 | 0.83 | 0.66 | 1.04 | 0.1099 | 0.92 | 0.74 | 1.15 | 0.4564 | 1.28 | 1.02 | 1.62 | 0.035* | 1.14 | 0.89 | 1.47 | 0.3059 |
|  | 3 | 1.20 | 0.94 | 1.53 | 0.1536 | 1.04 | 0.83 | 1.30 | 0.7403 | 1.00 | 0.80 | 1.24 | 0.9765 | 1.72 | 1.36 | 2.16 | <.0001* | 1.10 | 0.85 | 1.43 | 0.4691 |
|  | 4 | 1.54 | 1.19 | 1.98 | 0.0009* | 0.94 | 0.75 | 1.18 | 0.5964 | 1.07 | 0.86 | 1.34 | 0.5436 | 2.37 | 1.86 | 3.01 | <.0001* | 1.03 | 0.80 | 1.33 | 0.8275 |
| 1-month | 2 | 0.97 | 0.77 | 1.23 | 0.808 | 0.90 | 0.72 | 1.11 | 0.3169 | 0.88 | 0.72 | 1.09 | 0.2334 | 1.36 | 1.08 | 1.71 | 0.0078* | 1.10 | 0.86 | 1.39 | 0.4591 |
|  | 3 | 1.22 | 0.97 | 1.54 | 0.0955 | 1.12 | 0.90 | 1.38 | 0.3042 | 0.96 | 0.78 | 1.18 | 0.7119 | 1.74 | 1.39 | 2.17 | <.0001* | 0.95 | 0.74 | 1.21 | 0.6666 |
|  | 4 | 1.51 | 1.19 | 1.90 | 0.0006* | 0.99 | 0.80 | 1.24 | 0.9575 | 1.09 | 0.88 | 1.34 | 0.4327 | 2.29 | 1.83 | 2.88 | <.0001* | 1.03 | 0.81 | 1.31 | 0.8269 |
|  |  | **TNFα** | | | | **sTNFR2** | | | | **BAFF** | | | | **CCL2** | | | | **CCL17** | | | |
|  | **Cytokine quartile** | **OR** | **L** | **U** | **P value** | **OR** | **L** | **U** | **P value** | **OR** | **L** | **U** | **P value** | **OR** | **L** | **U** | **P value** | **OR** | **L** | **U** | **P value** |
| 12-month | 2 | 1.00 | 0.80 | 1.25 | 0.9878 | 1.36 | 1.09 | 1.70 | 0.0075 | 0.98 | 0.79 | 1.22 | 0.8664 | 0.98 | 0.78 | 1.22 | 0.8383 | 0.99 | 0.79 | 1.23 | 0.9126 |
|  | 3 | 1.00 | 0.80 | 1.25 | 0.9753 | 1.65 | 1.31 | 2.07 | <.0001* | 1.14 | 0.91 | 1.42 | 0.2467 | 1.06 | 0.85 | 1.33 | 0.5984 | 1.06 | 0.85 | 1.32 | 0.6161 |
|  | 4 | 1.24 | 0.99 | 1.55 | 0.0565 | 2.00 | 1.57 | 2.55 | <.0001* | 1.11 | 0.89 | 1.39 | 0.3436 | 1.33 | 1.07 | 1.67 | 0.0118* | 1.07 | 0.85 | 1.33 | 0.5804 |
| 3-month | 2 | 0.85 | 0.68 | 1.06 | 0.1565 | 1.30 | 1.03 | 1.64 | 0.0252 | 1.23 | 0.98 | 1.54 | 0.071 | 0.88 | 0.70 | 1.10 | 0.2525 | 0.94 | 0.75 | 1.17 | 0.5659 |
|  | 3 | 0.99 | 0.80 | 1.24 | 0.9463 | 2.07 | 1.64 | 2.62 | <.0001* | 1.55 | 1.24 | 1.95 | 0.0001* | 0.93 | 0.74 | 1.17 | 0.531 | 1.12 | 0.90 | 1.40 | 0.3171 |
|  | 4 | 1.09 | 0.88 | 1.37 | 0.4278 | 2.08 | 1.63 | 2.65 | <.0001* | 1.37 | 1.10 | 1.72 | 0.006 | 1.29 | 1.03 | 1.62 | 0.0246 | 1.09 | 0.87 | 1.35 | 0.4607 |
| 1-month | 2 | 0.86 | 0.70 | 1.06 | 0.1512 | 1.35 | 1.08 | 1.69 | 0.009 | 1.28 | 1.04 | 1.59 | 0.0219 | 1.07 | 0.87 | 1.33 | 0.5137 | 0.89 | 0.72 | 1.10 | 0.2768 |
|  | 3 | 1.04 | 0.84 | 1.28 | 0.7257 | 2.06 | 1.65 | 2.57 | <.0001* | 1.59 | 1.29 | 1.97 | <.0001* | 1.17 | 0.95 | 1.45 | 0.1407 | 1.10 | 0.89 | 1.35 | 0.3812 |
|  | 4 | 1.12 | 0.91 | 1.38 | 0.2724 | 2.01 | 1.59 | 2.53 | <.0001* | 1.35 | 1.09 | 1.67 | 0.0059 | 1.46 | 1.18 | 1.80 | 0.0004* | 0.97 | 0.79 | 1.19 | 0.7518 |
|  |  | **sCD14** | | | | **sCD25** | | | | **sCD27** | | | | **sCD163** | | | | **sgp130** | | | |
|  | **Cytokine quartile** | **OR** | **L** | **U** | **P value** | **OR** | **L** | **U** | **P value** | **OR** | **L** | **U** | **P value** | **OR** | **L** | **U** | **P value** | **OR** | **L** | **U** | **P value** |
| 12-month | 2 | 1.07 | 0.86 | 1.33 | 0.5491 | 1.16 | 0.93 | 1.45 | 0.1884 | 1.04 | 0.84 | 1.30 | 0.7273 | 1.23 | 0.99 | 1.54 | 0.0654 | 1.33 | 1.07 | 1.65 | 0.0113 |
|  | 3 | 1.00 | 0.80 | 1.25 | 0.992 | 1.09 | 0.87 | 1.35 | 0.4723 | 1.12 | 0.90 | 1.40 | 0.3127 | 1.23 | 0.98 | 1.54 | 0.0699 | 1.31 | 1.05 | 1.63 | 0.016 |
|  | 4 | 1.14 | 0.91 | 1.42 | 0.2605 | 1.31 | 1.05 | 1.64 | 0.0184 | 1.14 | 0.91 | 1.43 | 0.2687 | 1.25 | 0.99 | 1.57 | 0.056 | 1.43 | 1.14 | 1.79 | 0.002* |
| 3-month | 2 | 1.01 | 0.81 | 1.26 | 0.9035 | 1.24 | 0.99 | 1.55 | 0.058 | 1.19 | 0.95 | 1.50 | 0.1325 | 0.84 | 0.67 | 1.05 | 0.1263 | 1.75 | 1.38 | 2.23 | <.0001* |
|  | 3 | 1.17 | 0.93 | 1.46 | 0.1781 | 1.22 | 0.98 | 1.53 | 0.0756 | 1.58 | 1.26 | 1.98 | <.0001* | 0.91 | 0.73 | 1.13 | 0.3808 | 2.10 | 1.65 | 2.66 | <.0001* |
|  | 4 | 1.39 | 1.12 | 1.74 | 0.0035* | 1.27 | 1.01 | 1.59 | 0.0409 | 1.81 | 1.43 | 2.29 | <.0001* | 0.90 | 0.72 | 1.13 | 0.3604 | 3.14 | 2.46 | 4.02 | <.0001* |
| 1-month | 2 | 1.10 | 0.90 | 1.36 | 0.3567 | 1.19 | 0.97 | 1.47 | 0.0981 | 1.24 | 1.00 | 1.54 | 0.0556 | 0.93 | 0.76 | 1.15 | 0.5204 | 1.49 | 1.20 | 1.86 | 0.0004* |
|  | 3 | 1.11 | 0.90 | 1.37 | 0.321 | 1.23 | 1.00 | 1.52 | 0.0506 | 1.63 | 1.31 | 2.01 | <.0001* | 1.01 | 0.82 | 1.25 | 0.9009 | 1.77 | 1.42 | 2.21 | <.0001* |
|  | 4 | 1.38 | 1.12 | 1.70 | 0.0026* | 1.22 | 0.99 | 1.51 | 0.069 | 1.70 | 1.36 | 2.12 | <.0001* | 0.92 | 0.75 | 1.14 | 0.4519 | 2.22 | 1.78 | 2.77 | <.0001* |
| OR. Odds ratio; L. Lower limit of the 95% confidence interval; U. Upper limit of the 95% confidence interval; *. Association was significant after Bonferroni correction when not adjusted for other pollutants | | | | | | | | | | | | | | | | | | | | | |

Supplemental Table S5. Multivariable ordinal logistic regressions for the long-term (12-months) and short-term (3-months and 1-month) associations of nitrogen dioxide (NO_2_) exposure with the 15 tested cytokines. Immune markers are reported as quartiles (reference is Quartile 1), and associations are scaled by IQR.

|  |  | **IL-1β** | | | | **IL-6** | | | | **sIL-6Rα** | | | | **IL-8** | | | | **IL-10** | | | |
| --- | --- | --- | --- | --- | --- | --- | --- | --- | --- | --- | --- | --- | --- | --- | --- | --- | --- | --- | --- | --- | --- |
|  | **Cytokine quartile** | **OR** | **L** | **U** | **P value** | **OR** | **L** | **U** | **P value** | **OR** | **L** | **U** | **P value** | **OR** | **L** | **U** | **P value** | **OR** | **L** | **U** | **P value** |
| 12-month | 2 | 0.99 | 0.62 | 1.58 | 0.9557* | 1.36 | 0.87 | 2.11 | 0.1774 | 1.19 | 0.77 | 1.84 | 0.4397 | 0.93 | 0.60 | 1.43 | 0.7418 | 0.95 | 0.58 | 1.57 | 0.8444 |
|  | 3 | 1.34 | 0.84 | 2.16 | 0.2248* | 1.16 | 0.74 | 1.81 | 0.5206 | 0.83 | 0.53 | 1.28 | 0.3864 | 0.87 | 0.56 | 1.34 | 0.5197 | 1.63 | 0.98 | 2.72 | 0.0615 |
|  | 4 | 1.59 | 0.97 | 2.60 | 0.0678* | 1.52 | 0.97 | 2.40 | 0.0705 | 0.98 | 0.63 | 1.52 | 0.9311 | 1.40 | 0.88 | 2.23 | 0.1520 | 0.89 | 0.54 | 1.48 | 0.6551 |
| 3-month | 2 | 0.92 | 0.68 | 1.26 | 0.6150* | 1.26 | 0.95 | 1.68 | 0.1135 | 1.00 | 0.76 | 1.32 | 0.9896 | 0.93 | 0.70 | 1.23 | 0.6093 | 1.10 | 0.79 | 1.52 | 0.5834 |
|  | 3 | 1.33 | 0.97 | 1.80 | 0.0732* | 1.26 | 0.94 | 1.68 | 0.1256 | 0.98 | 0.75 | 1.30 | 0.9008 | 0.91 | 0.69 | 1.21 | 0.5309* | 1.65 | 1.20 | 2.29 | 0.0024 |
|  | 4 | 1.31 | 0.96 | 1.80 | 0.0875 | 1.28 | 0.95 | 1.71 | 0.1010 | 0.99 | 0.75 | 1.31 | 0.9248 | 1.41 | 1.06 | 1.88 | 0.0193* | 1.00 | 0.73 | 1.39 | 0.9825 |
| 1-month | 2 | 0.98 | 0.73 | 1.31 | 0.8834* | 1.15 | 0.88 | 1.51 | 0.3077 | 1.03 | 0.80 | 1.34 | 0.8073 | 0.92 | 0.72 | 1.19 | 0.5402* | 1.04 | 0.77 | 1.42 | 0.7863 |
|  | 3 | 1.28 | 0.95 | 1.71 | 0.1033 | 1.15 | 0.87 | 1.51 | 0.3307 | 1.01 | 0.78 | 1.32 | 0.9225 | 0.81 | 0.62 | 1.05 | 0.1100* | 1.45 | 1.07 | 1.96 | 0.0171 |
|  | 4 | 1.30 | 0.96 | 1.75 | 0.0885 | 1.32 | 1.01 | 1.74 | 0.0453 | 1.18 | 0.90 | 1.54 | 0.2224 | 1.05 | 0.80 | 1.38 | 0.7235* | 1.16 | 0.85 | 1.57 | 0.3556 |
|  |  | **TNFα** | | | | **sTNFR2** | | | | **BAFF** | | | | **CCL2** | | | | **CCL17** | | | |
|  | **Cytokine quartile** | **OR** | **L** | **U** | **P value** | **OR** | **L** | **U** | **P value** | **OR** | **L** | **U** | **P value** | **OR** | **L** | **U** | **P value** | **OR** | **L** | **U** | **P value** |
| 12-month | 2 | 0.91 | 0.59 | 1.40 | 0.6731 | 0.93 | 0.60 | 1.43 | 0.7270 | 1.07 | 0.69 | 1.65 | 0.7638 | 0.78 | 0.50 | 1.20 | 0.2588 | 0.86 | 0.55 | 1.32 | 0.4833 |
|  | 3 | 1.58 | 1.03 | 2.45 | 0.0381* | 1.21 | 0.78 | 1.89 | 0.3903 | 1.12 | 0.73 | 1.73 | 0.6111 | 0.89 | 0.58 | 1.38 | 0.6068 | 0.88 | 0.57 | 1.35 | 0.5468 |
|  | 4 | 1.26 | 0.82 | 1.96 | 0.2945 | 1.35 | 0.84 | 2.16 | 0.2118 | 1.12 | 0.73 | 1.74 | 0.6002 | 0.87 | 0.56 | 1.36 | 0.5412* | 0.95 | 0.62 | 1.46 | 0.8102 |
| 3-month | 2 | 1.12 | 0.85 | 1.49 | 0.4255 | 1.04 | 0.79 | 1.37 | 0.8039 | 1.04 | 0.79 | 1.38 | 0.7636 | 1.01 | 0.76 | 1.33 | 0.9670* | 0.93 | 0.70 | 1.23 | 0.6103 |
|  | 3 | 1.58 | 1.19 | 2.10 | 0.0015* | 1.14 | 0.86 | 1.52 | 0.3630 | 1.02 | 0.77 | 1.35 | 0.8731 | 1.08 | 0.82 | 1.43 | 0.5810 | 0.94 | 0.71 | 1.24 | 0.6543 |
|  | 4 | 1.63 | 1.22 | 2.17 | 0.0008 | 1.13 | 0.84 | 1.53 | 0.4271 | 1.03 | 0.78 | 1.36 | 0.8494 | 0.90 | 0.68 | 1.20 | 0.4873 | 1.20 | 0.91 | 1.58 | 0.1971 |
| 1-month | 2 | 1.19 | 0.92 | 1.56 | 0.1915 | 1.15 | 0.89 | 1.49 | 0.2863 | 1.26 | 0.97 | 1.64 | 0.0789 | 1.21 | 0.94 | 1.57 | 0.1462 | 0.92 | 0.71 | 1.20 | 0.5319 |
|  | 3 | 1.56 | 1.19 | 2.02 | 0.0010 | 1.18 | 0.90 | 1.55 | 0.2301 | 1.17 | 0.90 | 1.53 | 0.2372 | 1.14 | 0.88 | 1.48 | 0.3207 | 0.95 | 0.73 | 1.24 | 0.7096 |
|  | 4 | 1.40 | 1.07 | 1.83 | 0.0143 | 1.30 | 0.98 | 1.72 | 0.0741 | 1.16 | 0.89 | 1.51 | 0.2721 | 0.98 | 0.75 | 1.28 | 0.8884 | 1.09 | 0.84 | 1.41 | 0.5089 |
|  |  | **sCD14** | | | | **sCD25** | | | | **sCD27** | | | | **sCD163** | | | | **sgp130** | | | |
|  | **Cytokine quartile** | **OR** | **L** | **U** | **P value** | **OR** | **L** | **U** | **P value** | **OR** | **L** | **U** | **P value** | **OR** | **L** | **U** | **P value** | **OR** | **L** | **U** | **P value** |
| 12-month | 2 | 0.87 | 0.56 | 1.35 | 0.5416 | 1.12 | 0.73 | 1.72 | 0.6019 | 0.88 | 0.57 | 1.36 | 0.5767* | 1.18 | 0.76 | 1.81 | 0.4659 | 0.78 | 0.51 | 1.22 | 0.2776* |
|  | 3 | 0.89 | 0.57 | 1.37 | 0.5919 | 1.26 | 0.81 | 1.95 | 0.3026 | 1.15 | 0.75 | 1.78 | 0.5215 | 1.57 | 1.01 | 2.43 | 0.0454 | 0.93 | 0.60 | 1.45 | 0.7562* |
|  | 4 | 0.82 | 0.53 | 1.27 | 0.3707 | 1.22 | 0.78 | 1.90 | 0.3855 | 1.59 | 1.01 | 2.49 | 0.0451 | 1.36 | 0.87 | 2.13 | 0.1716 | 0.98 | 0.63 | 1.52 | 0.9369* |
| 3-month | 2 | 0.90 | 0.69 | 1.19 | 0.4578 | 1.24 | 0.94 | 1.63 | 0.1352 | 0.80 | 0.61 | 1.06 | 0.1164* | 1.18 | 0.89 | 1.56 | 0.2599 | 0.75 | 0.57 | 0.99 | 0.0391* |
|  | 3 | 0.91 | 0.69 | 1.20 | 0.5162 | 1.26 | 0.95 | 1.66 | 0.1114 | 0.91 | 0.69 | 1.21 | 0.5305 | 1.28 | 0.96 | 1.71 | 0.0883 | 0.82 | 0.62 | 1.08 | 0.1646* |
|  | 4 | 0.93 | 0.70 | 1.23 | 0.5885 | 1.26 | 0.95 | 1.68 | 0.1074 | 1.11 | 0.84 | 1.48 | 0.4685 | 1.34 | 1.01 | 1.79 | 0.0445 | 0.86 | 0.64 | 1.14 | 0.2820* |
| 1-month | 2 | 0.95 | 0.73 | 1.22 | 0.6720 | 1.20 | 0.93 | 1.56 | 0.1683 | 0.89 | 0.69 | 1.16 | 0.3985 | 1.21 | 0.93 | 1.58 | 0.1573 | 0.73 | 0.57 | 0.95 | 0.0178 |
|  | 3 | 0.94 | 0.72 | 1.22 | 0.6396 | 1.17 | 0.90 | 1.52 | 0.2427 | 1.03 | 0.79 | 1.34 | 0.8209 | 1.30 | 1.00 | 1.71 | 0.0538 | 0.73 | 0.57 | 0.95 | 0.0196 |
|  | 4 | 0.93 | 0.71 | 1.21 | 0.5758 | 1.26 | 0.96 | 1.64 | 0.0938 | 1.27 | 0.97 | 1.67 | 0.0806 | 1.32 | 1.00 | 1.73 | 0.0470 | 0.94 | 0.72 | 1.23 | 0.6656 |
| OR. Odds ratio; L. Lower limit of the 95% confidence interval; U. Upper limit of the 95% confidence interval; *. Association was significant after Bonferroni correction when not adjusted for other pollutants | | | | | | | | | | | | | | | | | | | | | |

Supplemental Table S6. Multivariable ordinal logistic regressions for the long-term (12-months) and short-term (3-months and 1-month) associations of particulate matter less than 0.1µm (PM0.1) exposure with the 15 tested cytokines. Immune markers are reported as quartiles (reference is Quartile 1), and associations are scaled by IQR.

|  |  | **IL-1β** | | | | **IL-6** | | | | **sIL-6Rα** | | | | **IL-8** | | | | **IL-10** | | | |
| --- | --- | --- | --- | --- | --- | --- | --- | --- | --- | --- | --- | --- | --- | --- | --- | --- | --- | --- | --- | --- | --- |
|  | **Cytokine quartile** | **OR** | **L** | **U** | **P value** | **OR** | **L** | **U** | **P value** | **OR** | **L** | **U** | **P value** | **OR** | **L** | **U** | **P value** | **OR** | **L** | **U** | **P value** |
| 12-month | 2 | 0.99 | 0.78 | 1.24 | 0.9003 | 1.11 | 0.89 | 1.38 | 0.3464 | 0.94 | 0.76 | 1.17 | 0.6042 | 0.99 | 0.81 | 1.22 | 0.9371 | 0.95 | 0.74 | 1.22 | 0.6618 |
|  | 3 | 0.86 | 0.68 | 1.09 | 0.2074 | 0.98 | 0.78 | 1.23 | 0.8505 | 1.19 | 0.96 | 1.46 | 0.1102 | 0.95 | 0.77 | 1.17 | 0.6342 | 0.78 | 0.61 | 1.02 | 0.0658 |
|  | 4 | 0.70 | 0.54 | 0.90 | 0.0057 | 0.98 | 0.78 | 1.23 | 0.8597 | 0.93 | 0.75 | 1.16 | 0.5285 | 0.57 | 0.45 | 0.72 | <.0001 | 1.01 | 0.79 | 1.29 | 0.9429 |
| 3-month | 2 | 0.88 | 0.70 | 1.11 | 0.2722 | 1.10 | 0.89 | 1.36 | 0.4011 | 0.98 | 0.79 | 1.20 | 0.8195 | 1.08 | 0.89 | 1.32 | 0.4407 | 0.86 | 0.68 | 1.10 | 0.2425 |
|  | 3 | 0.69 | 0.54 | 0.88 | 0.0028 | 0.97 | 0.78 | 1.21 | 0.7888 | 1.04 | 0.84 | 1.27 | 0.7476 | 0.96 | 0.78 | 1.18 | 0.6750 | 0.71 | 0.55 | 0.91 | 0.0079 |
|  | 4 | 0.64 | 0.49 | 0.82 | 0.0004 | 1.02 | 0.82 | 1.28 | 0.8593 | 0.95 | 0.77 | 1.17 | 0.6258 | 0.70 | 0.56 | 0.88 | 0.0022* | 0.93 | 0.73 | 1.18 | 0.5233 |
| 1-month | 2 | 0.86 | 0.70 | 1.04 | 0.1244 | 1.09 | 0.91 | 1.31 | 0.3515 | 1.03 | 0.86 | 1.24 | 0.7187 | 0.95 | 0.80 | 1.13 | 0.5662 | 0.92 | 0.75 | 1.13 | 0.4242 |
|  | 3 | 0.71 | 0.58 | 0.89 | 0.0025 | 1.00 | 0.82 | 1.21 | 0.9735 | 1.04 | 0.87 | 1.25 | 0.6630 | 0.91 | 0.76 | 1.09 | 0.3215 | 0.81 | 0.66 | 1.01 | 0.0622 |
|  | 4 | 0.66 | 0.52 | 0.83 | 0.0004 | 0.90 | 0.74 | 1.10 | 0.2933 | 0.80 | 0.66 | 0.98 | 0.0333 | 0.80 | 0.66 | 0.98 | 0.0312 | 0.83 | 0.66 | 1.03 | 0.0854 |
|  |  | **TNFα** | | | | **sTNFR2** | | | | **BAFF** | | | | **CCL2** | | | | **CCL17** | | | |
|  | **Cytokine quartile** | **OR** | **L** | **U** | **P value** | **OR** | **L** | **U** | **P value** | **OR** | **L** | **U** | **P value** | **OR** | **L** | **U** | **P value** | **OR** | **L** | **U** | **P value** |
| 12-month | 2 | 1.05 | 0.85 | 1.30 | 0.6628 | 0.96 | 0.78 | 1.18 | 0.7102 | 1.06 | 0.86 | 1.30 | 0.6069 | 1.26 | 1.02 | 1.57 | 0.0356* | 0.92 | 0.74 | 1.14 | 0.4345 |
|  | 3 | 0.99 | 0.80 | 1.22 | 0.8993 | 0.80 | 0.65 | 1.00 | 0.0463 | 0.89 | 0.72 | 1.10 | 0.2883 | 1.19 | 0.95 | 1.48 | 0.1246 | 0.93 | 0.75 | 1.15 | 0.5009 |
|  | 4 | 0.98 | 0.79 | 1.21 | 0.8246 | 0.69 | 0.54 | 0.87 | 0.0019 | 0.94 | 0.75 | 1.16 | 0.5504 | 1.10 | 0.88 | 1.38 | 0.3870* | 1.00 | 0.81 | 1.24 | 0.9949 |
| 3-month | 2 | 1.07 | 0.87 | 1.31 | 0.5364 | 0.96 | 0.78 | 1.17 | 0.6872 | 1.24 | 1.01 | 1.53 | 0.0380* | 1.31 | 1.05 | 1.62 | 0.0158* | 1.01 | 0.82 | 1.25 | 0.9026 |
|  | 3 | 1.02 | 0.82 | 1.25 | 0.8911 | 0.80 | 0.64 | 1.00 | 0.0458 | 1.01 | 0.82 | 1.26 | 0.9000 | 1.42 | 1.15 | 1.77 | 0.0014* | 0.94 | 0.76 | 1.16 | 0.5321 |
|  | 4 | 0.87 | 0.70 | 1.08 | 0.2104 | 0.83 | 0.66 | 1.04 | 0.1091 | 1.05 | 0.85 | 1.30 | 0.6751 | 1.30 | 1.04 | 1.63 | 0.0201* | 0.91 | 0.74 | 1.13 | 0.3942 |
| 1-month | 2 | 1.00 | 0.83 | 1.19 | 0.9548 | 0.85 | 0.71 | 1.01 | 0.0575 | 1.06 | 0.89 | 1.26 | 0.5051 | 0.98 | 0.81 | 1.18 | 0.7924 | 1.12 | 0.93 | 1.34 | 0.2516 |
|  | 3 | 0.93 | 0.78 | 1.12 | 0.4441 | 0.79 | 0.65 | 0.96 | 0.0151 | 0.90 | 0.74 | 1.09 | 0.2761 | 1.10 | 0.92 | 1.32 | 0.2901 | 0.99 | 0.81 | 1.20 | 0.9175 |
|  | 4 | 0.83 | 0.68 | 1.01 | 0.0568 | 0.73 | 0.59 | 0.89 | 0.0021 | 0.94 | 0.78 | 1.13 | 0.5104 | 1.09 | 0.90 | 1.31 | 0.3810 | 1.12 | 0.93 | 1.35 | 0.2242 |
|  |  | **sCD14** | | | | **sCD25** | | | | **sCD27** | | | | **sCD163** | | | | **sgp130** | | | |
|  | **Cytokine quartile** | **OR** | **L** | **U** | **P value** | **OR** | **L** | **U** | **P value** | **OR** | **L** | **U** | **P value** | **OR** | **L** | **U** | **P value** | **OR** | **L** | **U** | **P value** |
| 12-month | 2 | 0.97 | 0.79 | 1.20 | 0.7783 | 0.98 | 0.79 | 1.21 | 0.8276 | 0.99 | 0.80 | 1.21 | 0.9064 | 1.01 | 0.82 | 1.24 | 0.9501 | 0.93 | 0.76 | 1.15 | 0.5105 |
|  | 3 | 0.85 | 0.68 | 1.05 | 0.1244 | 0.99 | 0.80 | 1.22 | 0.9148 | 0.88 | 0.71 | 1.09 | 0.2352 | 0.94 | 0.76 | 1.16 | 0.5551 | 0.84 | 0.68 | 1.04 | 0.1111 |
|  | 4 | 0.79 | 0.64 | 0.99 | 0.0383 | 0.82 | 0.66 | 1.03 | 0.0843 | 0.72 | 0.58 | 0.91 | 0.0054 | 0.90 | 0.72 | 1.12 | 0.3561 | 0.76 | 0.61 | 0.94 | 0.0129 |
| 3-month | 2 | 1.09 | 0.89 | 1.33 | 0.4225 | 1.03 | 0.83 | 1.26 | 0.8138 | 1.03 | 0.84 | 1.27 | 0.7540 | 1.15 | 0.93 | 1.42 | 0.1962 | 1.02 | 0.83 | 1.25 | 0.8868 |
|  | 3 | 0.93 | 0.75 | 1.15 | 0.4990 | 1.08 | 0.88 | 1.33 | 0.4520 | 1.03 | 0.83 | 1.26 | 0.8089 | 1.19 | 0.96 | 1.48 | 0.1070 | 1.01 | 0.82 | 1.25 | 0.9154 |
|  | 4 | 0.89 | 0.72 | 1.11 | 0.2946 | 0.91 | 0.73 | 1.13 | 0.4018 | 0.85 | 0.68 | 1.06 | 0.1522 | 1.02 | 0.82 | 1.27 | 0.8779 | 0.83 | 0.66 | 1.04 | 0.0986* |
| 1-month | 2 | 0.95 | 0.80 | 1.13 | 0.5378 | 0.96 | 0.80 | 1.15 | 0.6677 | 0.95 | 0.80 | 1.13 | 0.5842 | 1.05 | 0.87 | 1.27 | 0.5968 | 0.98 | 0.82 | 1.17 | 0.8170 |
|  | 3 | 0.83 | 0.69 | 0.99 | 0.0428 | 1.01 | 0.85 | 1.21 | 0.8914 | 0.94 | 0.78 | 1.12 | 0.4637 | 1.11 | 0.92 | 1.34 | 0.2790 | 1.02 | 0.86 | 1.22 | 0.8000 |
|  | 4 | 0.85 | 0.70 | 1.02 | 0.0846 | 0.91 | 0.75 | 1.10 | 0.3358 | 0.76 | 0.62 | 0.93 | 0.0065 | 1.00 | 0.82 | 1.22 | 0.9918 | 0.79 | 0.65 | 0.96 | 0.0181* |
| OR. Odds ratio; L. Lower limit of the 95% confidence interval; U. Upper limit of the 95% confidence interval; *. Association was significant after Bonferroni correction when not adjusted for other pollutants | | | | | | | | | | | | | | | | | | | | | |

Supplemental Table S7. Multivariable ordinal logistic regressions for the long-term (12-months) and short-term (3-months and 1-month) associations of particulate matter less than 2.5µm (PM2.5) exposure with the 15 tested cytokines. Immune markers are reported as quartiles (reference is Quartile 1), and associations are scaled by IQR.

|  |  | **IL-1β** | | | | **IL-6** | | | | **sIL-6Rα** | | | | **IL-8** | | | | **IL-10** | | | |
| --- | --- | --- | --- | --- | --- | --- | --- | --- | --- | --- | --- | --- | --- | --- | --- | --- | --- | --- | --- | --- | --- |
|  | **Cytokine quartile** | **OR** | **L** | **U** | **P value** | **OR** | **L** | **U** | **P value** | **OR** | **L** | **U** | **P value** | **OR** | **L** | **U** | **P value** | **OR** | **L** | **U** | **P value** |
| 12-month | 2 | 0.89 | 0.59 | 1.34 | 0.5699* | 0.93 | 0.63 | 1.37 | 0.7103 | 0.64 | 0.44 | 0.94 | 0.0224 | 1.05 | 0.72 | 1.53 | 0.8169 | 1.07 | 0.69 | 1.68 | 0.7546 |
|  | 3 | 0.72 | 0.47 | 1.10 | 0.1245* | 1.10 | 0.74 | 1.62 | 0.6511 | 1.05 | 0.72 | 1.53 | 0.8211 | 1.35 | 0.92 | 1.96 | 0.1234 | 0.84 | 0.53 | 1.31 | 0.4379 |
|  | 4 | 0.63 | 0.41 | 0.97 | 0.0371* | 0.81 | 0.54 | 1.21 | 0.2943 | 0.88 | 0.60 | 1.29 | 0.4976 | 0.85 | 0.57 | 1.26 | 0.4203 | 1.18 | 0.76 | 1.85 | 0.4608 |
| 3-month | 2 | 1.01 | 0.82 | 1.26 | 0.9024 | 0.94 | 0.77 | 1.16 | 0.5781 | 0.91 | 0.74 | 1.11 | 0.3364 | 0.94 | 0.77 | 1.14 | 0.5205 | 0.96 | 0.76 | 1.22 | 0.7632 |
|  | 3 | 0.78 | 0.63 | 0.98 | 0.0324 | 0.95 | 0.77 | 1.18 | 0.6445 | 0.97 | 0.80 | 1.18 | 0.7712 | 0.98 | 0.81 | 1.20 | 0.8758* | 0.76 | 0.59 | 0.97 | 0.0258 |
|  | 4 | 0.90 | 0.72 | 1.13 | 0.3562 | 0.92 | 0.74 | 1.14 | 0.4412 | 0.92 | 0.75 | 1.13 | 0.4443 | 0.78 | 0.63 | 0.96 | 0.0201* | 1.05 | 0.83 | 1.33 | 0.6755 |
| 1-month | 2 | 0.98 | 0.81 | 1.20 | 0.8702 | 1.04 | 0.86 | 1.26 | 0.7077 | 0.91 | 0.75 | 1.09 | 0.2816 | 0.92 | 0.77 | 1.11 | 0.3751 | 1.01 | 0.81 | 1.25 | 0.9577 |
|  | 3 | 0.72 | 0.59 | 0.89 | 0.0024 | 1.00 | 0.82 | 1.22 | 0.9843 | 0.95 | 0.79 | 1.14 | 0.5665 | 1.12 | 0.94 | 1.34 | 0.2175 | 0.86 | 0.69 | 1.07 | 0.165 |
|  | 4 | 0.76 | 0.61 | 0.94 | 0.0113 | 0.86 | 0.70 | 1.05 | 0.1279 | 0.75 | 0.62 | 0.91 | 0.0041 | 0.83 | 0.68 | 1.02 | 0.0721 | 0.95 | 0.77 | 1.19 | 0.6652 |
|  |  | **TNFα** | | | | **sTNFR2** | | | | **BAFF** | | | | **CCL2** | | | | **CCL17** | | | |
|  | **Cytokine quartile** | **OR** | **L** | **U** | **P value** | **OR** | **L** | **U** | **P value** | **OR** | **L** | **U** | **P value** | **OR** | **L** | **U** | **P value** | **OR** | **L** | **U** | **P value** |
| 12-month | 2 | 1.27 | 0.87 | 1.85 | 0.2146 | 1.06 | 0.73 | 1.54 | 0.7689 | 1.21 | 0.83 | 1.77 | 0.3177 | 1.69 | 1.15 | 2.48 | 0.0078* | 1.16 | 0.79 | 1.70 | 0.4529 |
|  | 3 | 0.93 | 0.64 | 1.36 | 0.7109 | 0.97 | 0.66 | 1.43 | 0.8855 | 1.01 | 0.69 | 1.47 | 0.9797 | 1.29 | 0.88 | 1.89 | 0.1994 | 1.25 | 0.85 | 1.83 | 0.2594 |
|  | 4 | 0.97 | 0.66 | 1.43 | 0.8767 | 0.83 | 0.55 | 1.25 | 0.3675 | 0.93 | 0.64 | 1.37 | 0.7254 | 1.45 | 0.98 | 2.15 | 0.0599* | 1.19 | 0.81 | 1.74 | 0.3765 |
| 3-month | 2 | 0.98 | 0.81 | 1.20 | 0.8508 | 1.00 | 0.82 | 1.21 | 0.9739 | 1.13 | 0.93 | 1.37 | 0.2334 | 1.16 | 0.95 | 1.43 | 0.1472* | 1.01 | 0.82 | 1.24 | 0.9161 |
|  | 3 | 0.82 | 0.67 | 1.01 | 0.0582 | 0.97 | 0.78 | 1.19 | 0.7363 | 1.02 | 0.83 | 1.25 | 0.861 | 0.98 | 0.79 | 1.20 | 0.8207 | 1.07 | 0.87 | 1.31 | 0.5219 |
|  | 4 | 0.70 | 0.57 | 0.87 | 0.0012 | 0.98 | 0.79 | 1.22 | 0.8465 | 1.02 | 0.83 | 1.25 | 0.864 | 1.23 | 0.99 | 1.51 | 0.0566 | 0.97 | 0.80 | 1.19 | 0.7972 |
| 1-month | 2 | 0.92 | 0.77 | 1.11 | 0.4005 | 0.86 | 0.72 | 1.04 | 0.1139 | 0.94 | 0.79 | 1.13 | 0.4965 | 1.02 | 0.85 | 1.23 | 0.8099 | 1.04 | 0.87 | 1.26 | 0.6505 |
|  | 3 | 0.85 | 0.71 | 1.02 | 0.0793 | 0.87 | 0.72 | 1.05 | 0.1376 | 0.84 | 0.70 | 1.02 | 0.0726 | 0.97 | 0.81 | 1.17 | 0.7738 | 1.03 | 0.85 | 1.25 | 0.746 |
|  | 4 | 0.77 | 0.63 | 0.93 | 0.0074 | 0.80 | 0.65 | 0.98 | 0.0271 | 0.88 | 0.73 | 1.06 | 0.1774 | 1.11 | 0.92 | 1.35 | 0.263 | 1.05 | 0.87 | 1.26 | 0.629 |
|  |  | **sCD14** | | | | **sCD25** | | | | **sCD27** | | | | **sCD163** | | | | **sgp130** | | | |
|  | **Cytokine quartile** | **OR** | **L** | **U** | **P value** | **OR** | **L** | **U** | **P value** | **OR** | **L** | **U** | **P value** | **OR** | **L** | **U** | **P value** | **OR** | **L** | **U** | **P value** |
| 12-month | 2 | 1.06 | 0.72 | 1.55 | 0.7776 | 1.05 | 0.72 | 1.53 | 0.8032 | 0.94 | 0.64 | 1.36 | 0.7257 | 0.86 | 0.59 | 1.26 | 0.4442 | 0.97 | 0.66 | 1.42 | 0.8678 |
|  | 3 | 1.10 | 0.75 | 1.62 | 0.6105 | 0.92 | 0.63 | 1.34 | 0.6578 | 0.85 | 0.58 | 1.24 | 0.388 | 0.66 | 0.45 | 0.97 | 0.0364 | 0.83 | 0.57 | 1.21 | 0.3365 |
|  | 4 | 1.19 | 0.81 | 1.74 | 0.3844 | 0.90 | 0.61 | 1.32 | 0.5759 | 0.78 | 0.53 | 1.16 | 0.2192 | 0.78 | 0.53 | 1.15 | 0.204 | 0.99 | 0.67 | 1.44 | 0.9414 |
| 3-month | 2 | 1.00 | 0.82 | 1.22 | 0.9997 | 0.89 | 0.73 | 1.09 | 0.268 | 1.03 | 0.84 | 1.25 | 0.7797 | 0.97 | 0.79 | 1.19 | 0.7544 | 1.11 | 0.91 | 1.35 | 0.3133* |
|  | 3 | 0.97 | 0.80 | 1.19 | 0.7919 | 0.87 | 0.71 | 1.07 | 0.1814 | 1.01 | 0.83 | 1.23 | 0.9405 | 0.89 | 0.72 | 1.09 | 0.2466 | 0.98 | 0.79 | 1.20 | 0.8194* |
|  | 4 | 0.93 | 0.76 | 1.14 | 0.4731 | 0.85 | 0.70 | 1.05 | 0.1292 | 0.92 | 0.75 | 1.14 | 0.4449 | 0.88 | 0.71 | 1.08 | 0.2267 | 1.08 | 0.88 | 1.33 | 0.4469* |
| 1-month | 2 | 0.95 | 0.80 | 1.14 | 0.5869 | 0.92 | 0.77 | 1.10 | 0.3653 | 0.89 | 0.74 | 1.07 | 0.2046 | 0.95 | 0.79 | 1.15 | 0.5958 | 1.07 | 0.89 | 1.28 | 0.4717 |
|  | 3 | 0.93 | 0.78 | 1.12 | 0.4629 | 0.92 | 0.76 | 1.10 | 0.3619 | 0.86 | 0.71 | 1.03 | 0.0956 | 0.86 | 0.71 | 1.04 | 0.1279 | 1.01 | 0.84 | 1.21 | 0.9416* |
|  | 4 | 0.91 | 0.76 | 1.10 | 0.3452 | 0.82 | 0.68 | 0.99 | 0.0383 | 0.79 | 0.65 | 0.96 | 0.0152 | 0.88 | 0.73 | 1.07 | 0.2016 | 0.97 | 0.80 | 1.16 | 0.716 |
| OR. Odds ratio; L. Lower limit of the 95% confidence interval; U. Upper limit of the 95% confidence interval; *. Association was significant after Bonferroni correction when not adjusted for other pollutants | | | | | | | | | | | | | | | | | | | | | |

Supplemental Table S8. Multivariable ordinal logistic regressions for the long-term (12-months) and short-term (3-months and 1-month) associations of particulate matter less than 10µm (PM10) exposure with the 15 tested cytokines.

|  |  | **IL-1β** | | | | **IL-6** | | | | **sIL-6Rα** | | | | **IL-8** | | | | **IL-10** | | | |
| --- | --- | --- | --- | --- | --- | --- | --- | --- | --- | --- | --- | --- | --- | --- | --- | --- | --- | --- | --- | --- | --- |
|  | **Cytokine quartile** | **OR** | **L** | **U** | **P value** | **OR** | **L** | **U** | **P value** | **OR** | **L** | **U** | **P value** | **OR** | **L** | **U** | **P value** | **OR** | **L** | **U** | **P value** |
| 12-month | 2 | 1.01 | 0.74 | 1.39 | 0.9344* | 0.87 | 0.65 | 1.18 | 0.3749 | 0.82 | 0.61 | 1.10 | 0.1806 | 0.90 | 0.67 | 1.21 | 0.4912 | 1.07 | 0.76 | 1.51 | 0.6817 |
|  | 3 | 0.86 | 0.62 | 1.18 | 0.3474* | 0.93 | 0.69 | 1.25 | 0.6372 | 1.05 | 0.79 | 1.40 | 0.7303 | 1.13 | 0.84 | 1.51 | 0.4283 | 0.71 | 0.50 | 1.01 | 0.0594 |
|  | 4 | 0.56 | 0.40 | 0.78 | 0.0006 | 0.74 | 0.55 | 1.01 | 0.0587 | 1.06 | 0.79 | 1.42 | 0.6892 | 0.49 | 0.36 | 0.66 | <.0001 | 1.03 | 0.73 | 1.45 | 0.8846 |
| 3-month | 2 | 1.07 | 0.91 | 1.27 | 0.4242 | 0.94 | 0.80 | 1.10 | 0.4413 | 0.97 | 0.82 | 1.13 | 0.6582 | 0.89 | 0.76 | 1.04 | 0.1324* | 0.98 | 0.82 | 1.19 | 0.8686 |
|  | 3 | 0.89 | 0.74 | 1.06 | 0.1898 | 0.95 | 0.80 | 1.12 | 0.5276 | 1.01 | 0.87 | 1.18 | 0.8891 | 0.92 | 0.79 | 1.07 | 0.2823* | 0.76 | 0.62 | 0.93 | 0.0068 |
|  | 4 | 0.90 | 0.75 | 1.08 | 0.2520 | 0.87 | 0.73 | 1.03 | 0.1069 | 0.98 | 0.83 | 1.15 | 0.7684 | 0.64 | 0.53 | 0.77 | <.0001* | 1.01 | 0.84 | 1.22 | 0.8797 |
| 1-month | 2 | 1.04 | 0.88 | 1.22 | 0.6702 | 0.97 | 0.83 | 1.15 | 0.7523 | 0.95 | 0.81 | 1.11 | 0.5122 | 0.89 | 0.76 | 1.04 | 0.1397 | 0.99 | 0.82 | 1.19 | 0.8852 |
|  | 3 | 0.84 | 0.70 | 1.01 | 0.0576 | 0.98 | 0.83 | 1.15 | 0.7620 | 0.99 | 0.85 | 1.16 | 0.9081 | 1.04 | 0.89 | 1.21 | 0.6014 | 0.83 | 0.68 | 1.00 | 0.0478 |
|  | 4 | 0.82 | 0.68 | 0.99 | 0.0339 | 0.83 | 0.69 | 0.99 | 0.0344 | 0.86 | 0.72 | 1.01 | 0.0686 | 0.70 | 0.58 | 0.84 | 0.0002 | 0.93 | 0.77 | 1.12 | 0.4455 |
|  |  | **TNFα** | | | | **sTNFR2** | | | | **BAFF** | | | | **CCL2** | | | | **CCL17** | | | |
|  | **Cytokine quartile** | **OR** | **L** | **U** | **P value** | **OR** | **L** | **U** | **P value** | **OR** | **L** | **U** | **P value** | **OR** | **L** | **U** | **P value** | **OR** | **L** | **U** | **P value** |
| 12-month | 2 | 1.07 | 0.80 | 1.42 | 0.6716 | 1.02 | 0.76 | 1.37 | 0.8931 | 1.15 | 0.86 | 1.54 | 0.3547 | 1.34 | 0.99 | 1.81 | 0.0582* | 0.97 | 0.72 | 1.30 | 0.8291 |
|  | 3 | 0.85 | 0.64 | 1.14 | 0.2821 | 0.99 | 0.73 | 1.33 | 0.9279 | 1.13 | 0.84 | 1.51 | 0.4291 | 1.27 | 0.94 | 1.71 | 0.1202 | 0.99 | 0.74 | 1.33 | 0.9689 |
|  | 4 | 0.83 | 0.62 | 1.11 | 0.1986 | 0.87 | 0.63 | 1.19 | 0.3757 | 1.01 | 0.75 | 1.36 | 0.9618 | 1.24 | 0.92 | 1.68 | 0.1597* | 1.10 | 0.82 | 1.46 | 0.5262 |
| 3-month | 2 | 0.96 | 0.82 | 1.12 | 0.5969 | 1.00 | 0.85 | 1.16 | 0.9457 | 1.01 | 0.87 | 1.19 | 0.8635 | 1.06 | 0.90 | 1.24 | 0.4978* | 0.94 | 0.80 | 1.10 | 0.4354 |
|  | 3 | 0.85 | 0.73 | 1.00 | 0.0435 | 0.98 | 0.83 | 1.15 | 0.7563 | 1.03 | 0.87 | 1.21 | 0.7604 | 0.96 | 0.81 | 1.14 | 0.6476 | 0.99 | 0.84 | 1.16 | 0.9049 |
|  | 4 | 0.76 | 0.64 | 0.90 | 0.0014 | 0.95 | 0.80 | 1.13 | 0.5556 | 1.00 | 0.85 | 1.18 | 0.9658 | 1.15 | 0.97 | 1.35 | 0.1079 | 0.98 | 0.84 | 1.14 | 0.8035 |
| 1-month | 2 | 0.91 | 0.78 | 1.07 | 0.2508 | 0.93 | 0.79 | 1.08 | 0.3259 | 0.92 | 0.78 | 1.07 | 0.2801 | 0.98 | 0.84 | 1.15 | 0.8347 | 0.98 | 0.83 | 1.15 | 0.8188 |
|  | 3 | 0.85 | 0.72 | 0.99 | 0.0383 | 0.94 | 0.80 | 1.11 | 0.4452 | 0.95 | 0.81 | 1.12 | 0.5622 | 0.93 | 0.79 | 1.10 | 0.4117 | 1.00 | 0.85 | 1.18 | 0.9771 |
|  | 4 | 0.79 | 0.67 | 0.94 | 0.0063 | 0.86 | 0.72 | 1.03 | 0.1001 | 0.93 | 0.79 | 1.10 | 0.3914 | 1.07 | 0.91 | 1.26 | 0.4091 | 1.02 | 0.88 | 1.20 | 0.7806 |
|  |  | **sCD14** | | | | **sCD25** | | | | **sCD27** | | | | **sCD163** | | | | **sgp130** | | | |
|  | **Cytokine quartile** | **OR** | **L** | **U** | **P value** | **OR** | **L** | **U** | **P value** | **OR** | **L** | **U** | **P value** | **OR** | **L** | **U** | **P value** | **OR** | **L** | **U** | **P value** |
| 12-month | 2 | 1.02 | 0.76 | 1.37 | 0.9083 | 1.04 | 0.78 | 1.39 | 0.7846 | 0.97 | 0.73 | 1.30 | 0.8567 | 0.89 | 0.66 | 1.19 | 0.4184 | 1.08 | 0.81 | 1.46 | 0.5947 |
|  | 3 | 1.10 | 0.82 | 1.47 | 0.5380 | 0.94 | 0.70 | 1.27 | 0.7002 | 1.00 | 0.75 | 1.33 | 0.9822 | 0.78 | 0.58 | 1.05 | 0.0959 | 1.08 | 0.80 | 1.45 | 0.6322 |
|  | 4 | 1.29 | 0.96 | 1.73 | 0.0869 | 0.96 | 0.71 | 1.30 | 0.7932 | 0.93 | 0.69 | 1.26 | 0.6319 | 0.85 | 0.63 | 1.14 | 0.2810 | 1.30 | 0.97 | 1.74 | 0.0810 |
| 3-month | 2 | 0.98 | 0.84 | 1.15 | 0.8245 | 0.97 | 0.83 | 1.13 | 0.6829 | 0.99 | 0.85 | 1.16 | 0.9170 | 0.98 | 0.84 | 1.15 | 0.8267 | 1.07 | 0.91 | 1.25 | 0.4147* |
|  | 3 | 0.97 | 0.83 | 1.13 | 0.6939 | 0.93 | 0.79 | 1.10 | 0.3988 | 1.00 | 0.85 | 1.17 | 0.9639 | 0.94 | 0.80 | 1.11 | 0.4924 | 0.98 | 0.84 | 1.16 | 0.8352* |
|  | 4 | 0.98 | 0.83 | 1.15 | 0.7927 | 0.96 | 0.82 | 1.13 | 0.6423 | 0.92 | 0.78 | 1.08 | 0.2967 | 0.94 | 0.80 | 1.11 | 0.4916 | 1.10 | 0.93 | 1.29 | 0.2644* |
| 1-month | 2 | 0.98 | 0.84 | 1.14 | 0.7754 | 0.97 | 0.83 | 1.14 | 0.7200 | 0.93 | 0.79 | 1.09 | 0.3662 | 0.96 | 0.81 | 1.12 | 0.5740 | 1.09 | 0.93 | 1.27 | 0.2972 |
|  | 3 | 0.98 | 0.84 | 1.15 | 0.7990 | 0.97 | 0.83 | 1.14 | 0.7096 | 0.93 | 0.79 | 1.09 | 0.3699 | 0.91 | 0.78 | 1.08 | 0.2850 | 1.07 | 0.91 | 1.26 | 0.4098 |
|  | 4 | 1.01 | 0.86 | 1.19 | 0.9040 | 0.93 | 0.79 | 1.10 | 0.3870 | 0.86 | 0.73 | 1.02 | 0.0793 | 0.92 | 0.78 | 1.09 | 0.3216 | 1.09 | 0.93 | 1.28 | 0.2864 |
| OR. Odds ratio; L. Lower limit of the 95% confidence interval; U. Upper limit of the 95% confidence interval; *. Association was significant after Bonferroni correction when not adjusted for other pollutants | | | | | | | | | | | | | | | | | | | | | |

Supplemental Table S9. P-trends for the multivariable ordinal logistic regressions for the long-term (12-months) and short-term (3-months and 1-month) associations of air pollutant exposure with the 15 tested cytokines. Immune markers are reported as quartiles (reference is Quartile 1), and associations are scaled by IQR.

| Exposure | Time before blood draw | IL-1β | IL-8 | TNFα | sTNFR2 | CCL2 | sCD14 | sCD163 | IL-6 | sIL-6Rα | sgp130 | IL-10 | BAFF | sCD27 | CCL17 | sCD25 |
| --- | --- | --- | --- | --- | --- | --- | --- | --- | --- | --- | --- | --- | --- | --- | --- | --- |
| O_3_ | 12-month | <.0001 | <.0001 | 0.072 | <.0001 | 0.009 | 0.393 | 0.086 | 0.252 | 0.911 | 0.003 | 0.670 | 0.211 | 0.172 | 0.462 | 0.049 |
|  | 3-month | 0.0002 | <.0001 | 0.272 | <.0001 | 0.012 | 0.001 | 0.532 | 0.985 | 0.402 | <.0001 | 0.852 | 0.002 | <.0001 | 0.226 | 0.054 |
|  | 1-month | <.0001 | <.0001 | 0.138 | <.0001 | 0.0002 | 0.003 | 0.624 | 0.665 | 0.326 | <.0001 | 0.890 | 0.002 | <.0001 | 0.800 | 0.065 |
| NO_2_ | 12-month | 0.021 | 0.059 | 0.085 | 0.119 | 0.808 | 0.450 | 0.113 | 0.109 | 0.464 | 0.807 | 0.801 | 0.576 | 0.026 | 0.864 | 0.247 |
|  | 3-month | 0.027 | 0.038 | 0.0002 | 0.248 | 0.728 | 0.683 | 0.051 | 0.128 | 0.831 | 0.359 | 0.381 | 0.895 | 0.309 | 0.191 | 0.089 |
|  | 1-month | 0.032 | 0.906 | 0.005 | 0.051 | 0.879 | 0.621 | 0.060 | 0.060 | 0.349 | 0.518 | 0.114 | 0.379 | 0.053 | 0.484 | 0.092 |
| PM0.1 | 12-month | 0.002 | <.0001 | 0.681 | 0.001 | 0.556 | 0.018 | 0.309 | 0.664 | 0.823 | 0.006 | 0.721 | 0.341 | 0.004 | 0.998 | 0.115 |
|  | 3-month | <.0001 | 0.0009 | 0.218 | 0.035 | 0.015 | 0.152 | 0.767 | 0.954 | 0.919 | 0.064 | 0.263 | 0.862 | 0.150 | 0.273 | 0.498 |
|  | 1-month | <.0001 | 0.040 | 0.054 | 0.001 | 0.204 | 0.039 | 0.806 | 0.254 | 0.101 | 0.035 | 0.050 | 0.229 | 0.006 | 0.414 | 0.403 |
| PM2.5 | 12-month | 0.020 | 0.648 | 0.553 | 0.423 | 0.212 | 0.366 | 0.131 | 0.406 | 0.841 | 0.726 | 0.731 | 0.535 | 0.170 | 0.341 | 0.420 |
|  | 3-month | 0.134 | 0.024 | 0.001 | 0.702 | 0.255 | 0.419 | 0.180 | 0.470 | 0.610 | 0.947 | 0.845 | 0.882 | 0.281 | 0.920 | 0.112 |
|  | 1-month | 0.002 | 0.245 | 0.007 | 0.022 | 0.422 | 0.321 | 0.166 | 0.136 | 0.015 | 0.426 | 0.389 | 0.087 | 0.004 | 0.626 | 0.046 |
| PM10 | 12-month | 0.0003 | <.0001 | 0.101 | 0.486 | 0.298 | 0.067 | 0.223 | 0.078 | 0.310 | 0.097 | 0.571 | 0.985 | 0.686 | 0.470 | 0.607 |
|  | 3-month | 0.115 | <.0001 | 0.001 | 0.487 | 0.284 | 0.693 | 0.466 | 0.135 | 0.928 | 0.677 | 0.535 | 0.944 | 0.208 | 0.999 | 0.506 |
|  | 1-month | 0.015 | 0.003 | 0.007 | 0.103 | 0.636 | 0.950 | 0.334 | 0.049 | 0.131 | 0.428 | 0.202 | 0.480 | 0.044 | 0.675 | 0.366 |

Supplemental Table S10. Sensitivity analysis (removal of extreme pollutant estimates) of the multivariable ordinal logistic regressions for the long-term (12-months) and short-term (3-months and 1-month) associations of ozone (O_3_) exposure with the 15 tested cytokines. Immune markers are reported as quartiles (reference is Quartile 1), and associations are scaled by IQR.

|  |  | **IL-1β** | | | | **IL-6** | | | | **sIL-6Rα** | | | | **IL-8** | | | | **IL-10** | | | |
| --- | --- | --- | --- | --- | --- | --- | --- | --- | --- | --- | --- | --- | --- | --- | --- | --- | --- | --- | --- | --- | --- |
|  | **Cytokine quartile** | **OR** | **L** | **U** | **P value** | **OR** | **L** | **U** | **P value** | **OR** | **L** | **U** | **P value** | **OR** | **L** | **U** | **P value** | **OR** | **L** | **U** | **P value** |
| 12-month | 2 | 1.20 | 0.92 | 1.57 | 0.1864 | 0.96 | 0.74 | 1.25 | 0.7661 | 0.90 | 0.70 | 1.16 | 0.4215 | 1.47 | 1.12 | 1.93 | 0.0059 | 0.90 | 0.70 | 1.16 | 0.4229 |
|  | 3 | 1.55 | 1.18 | 2.03 | 0.0015 | 1.33 | 1.03 | 1.73 | 0.0317 | 1.00 | 0.78 | 1.30 | 0.9825 | 1.31 | 0.99 | 1.72 | 0.0584 | 1.08 | 0.84 | 1.40 | 0.553 |
|  | 4 | 2.57 | 1.95 | 3.37 | <.0001 | 1.20 | 0.93 | 1.57 | 0.168 | 0.82 | 0.63 | 1.06 | 0.1216 | 3.70 | 2.80 | 4.89 | <.0001 | 1.02 | 0.79 | 1.32 | 0.8755 |
| 3-month | 2 | 1.05 | 0.80 | 1.36 | 0.7339 | 0.80 | 0.61 | 1.04 | 0.0959 | 0.90 | 0.69 | 1.17 | 0.4346 | 1.09 | 0.83 | 1.44 | 0.5439 | 0.93 | 0.72 | 1.21 | 0.5862 |
|  | 3 | 1.17 | 0.89 | 1.53 | 0.2548 | 0.99 | 0.76 | 1.29 | 0.9487 | 0.93 | 0.71 | 1.21 | 0.5733 | 1.39 | 1.05 | 1.83 | 0.0217 | 1.09 | 0.83 | 1.42 | 0.5372 |
|  | 4 | 1.65 | 1.26 | 2.17 | 0.0003 | 0.87 | 0.66 | 1.14 | 0.3037 | 1.02 | 0.78 | 1.33 | 0.8672 | 1.92 | 1.45 | 2.55 | <.0001 | 0.94 | 0.72 | 1.23 | 0.6584 |
| 1-month | 2 | 1.09 | 0.83 | 1.45 | 0.5309 | 1.05 | 0.80 | 1.37 | 0.7526 | 0.98 | 0.75 | 1.29 | 0.8978 | 1.06 | 0.80 | 1.42 | 0.6713 | 0.87 | 0.66 | 1.14 | 0.2978 |
|  | 3 | 1.29 | 0.98 | 1.71 | 0.075 | 1.12 | 0.85 | 1.47 | 0.4121 | 0.85 | 0.65 | 1.11 | 0.2393 | 1.46 | 1.10 | 1.95 | 0.0097 | 1.06 | 0.81 | 1.39 | 0.6847 |
|  | 4 | 1.67 | 1.26 | 2.21 | 0.0004 | 0.95 | 0.72 | 1.26 | 0.7141 | 1.16 | 0.88 | 1.52 | 0.301 | 1.99 | 1.50 | 2.64 | <.0001 | 0.99 | 0.76 | 1.30 | 0.9569 |
|  |  | **TNFα** | | | | **sTNFR2** | | | | **BAFF** | | | | **CCL2** | | | | **CCL17** | | | |
|  | **Cytokine quartile** | **OR** | **L** | **U** | **P value** | **OR** | **L** | **U** | **P value** | **OR** | **L** | **U** | **P value** | **OR** | **L** | **U** | **P value** | **OR** | **L** | **U** | **P value** |
| 12-month | 2 | 1.00 | 0.77 | 1.30 | 0.9992 | 1.46 | 1.12 | 1.90 | 0.0046 | 0.97 | 0.75 | 1.26 | 0.8261 | 1.02 | 0.79 | 1.31 | 0.9092 | 0.95 | 0.74 | 1.23 | 0.6959 |
|  | 3 | 1.04 | 0.80 | 1.34 | 0.7909 | 1.84 | 1.41 | 2.41 | <.0001 | 1.15 | 0.89 | 1.48 | 0.29 | 1.09 | 0.85 | 1.41 | 0.4947 | 0.97 | 0.75 | 1.25 | 0.7869 |
|  | 4 | 1.32 | 1.02 | 1.71 | 0.0352 | 2.17 | 1.64 | 2.86 | <.0001 | 1.09 | 0.85 | 1.41 | 0.4966 | 1.40 | 1.08 | 1.80 | 0.01 | 0.99 | 0.76 | 1.27 | 0.9091 |
| 3-month | 2 | 0.81 | 0.62 | 1.06 | 0.1202 | 1.38 | 1.05 | 1.81 | 0.0232 | 1.28 | 0.98 | 1.68 | 0.0697 | 0.76 | 0.58 | 1.00 | 0.0516 | 0.89 | 0.69 | 1.17 | 0.4098 |
|  | 3 | 0.92 | 0.70 | 1.19 | 0.5078 | 2.23 | 1.69 | 2.96 | <.0001 | 1.59 | 1.22 | 2.09 | 0.0008 | 0.88 | 0.67 | 1.15 | 0.3519 | 1.04 | 0.80 | 1.36 | 0.7536 |
|  | 4 | 0.96 | 0.74 | 1.25 | 0.7866 | 2.12 | 1.59 | 2.84 | <.0001 | 1.52 | 1.16 | 2.00 | 0.0028 | 1.22 | 0.93 | 1.60 | 0.1473 | 1.11 | 0.85 | 1.45 | 0.4307 |
| 1-month | 2 | 0.77 | 0.59 | 1.02 | 0.0638 | 1.33 | 1.00 | 1.78 | 0.05 | 1.29 | 0.98 | 1.70 | 0.0666 | 0.92 | 0.70 | 1.22 | 0.5676 | 0.75 | 0.57 | 0.99 | 0.0395 |
|  | 3 | 1.10 | 0.83 | 1.44 | 0.508 | 2.69 | 2.00 | 3.62 | <.0001 | 1.63 | 1.23 | 2.15 | 0.0006 | 1.03 | 0.79 | 1.35 | 0.8136 | 1.00 | 0.76 | 1.32 | 0.98 |
|  | 4 | 0.97 | 0.74 | 1.28 | 0.8249 | 2.31 | 1.70 | 3.13 | <.0001 | 1.39 | 1.05 | 1.84 | 0.0212 | 1.39 | 1.06 | 1.82 | 0.0177 | 0.98 | 0.74 | 1.29 | 0.8633 |
|  |  | **sCD14** | | | | **sCD25** | | | | **sCD27** | | | | **sCD163** | | | | **sgp130** | | | |
|  | **Cytokine quartile** | **OR** | **L** | **U** | **P value** | **OR** | **L** | **U** | **P value** | **OR** | **L** | **U** | **P value** | **OR** | **L** | **U** | **P value** | **OR** | **L** | **U** | **P value** |
| 12-month | 2 | 1.12 | 0.87 | 1.43 | 0.3939 | 1.15 | 0.89 | 1.49 | 0.2772 | 1.05 | 0.81 | 1.35 | 0.7109 | 1.29 | 1.00 | 1.67 | 0.0526 | 1.40 | 1.09 | 1.80 | 0.0088 |
|  | 3 | 1.12 | 0.87 | 1.45 | 0.3715 | 1.00 | 0.77 | 1.29 | 0.9958 | 1.16 | 0.90 | 1.50 | 0.2659 | 1.22 | 0.94 | 1.57 | 0.1354 | 1.42 | 1.10 | 1.83 | 0.007 |
|  | 4 | 1.10 | 0.85 | 1.42 | 0.4579 | 1.25 | 0.96 | 1.62 | 0.0928 | 1.22 | 0.94 | 1.59 | 0.1364 | 1.17 | 0.91 | 1.52 | 0.2271 | 1.61 | 1.24 | 2.08 | 0.0003 |
| 3-month | 2 | 0.99 | 0.75 | 1.29 | 0.9122 | 1.12 | 0.86 | 1.47 | 0.4059 | 1.05 | 0.80 | 1.37 | 0.7314 | 0.86 | 0.65 | 1.12 | 0.2522 | 1.85 | 1.40 | 2.46 | <.0001 |
|  | 3 | 1.08 | 0.83 | 1.41 | 0.5806 | 1.09 | 0.84 | 1.42 | 0.5306 | 1.51 | 1.15 | 1.98 | 0.0033 | 0.90 | 0.69 | 1.16 | 0.405 | 2.36 | 1.77 | 3.14 | <.0001 |
|  | 4 | 1.37 | 1.05 | 1.80 | 0.0199 | 1.16 | 0.88 | 1.53 | 0.2798 | 1.58 | 1.20 | 2.09 | 0.0012 | 0.96 | 0.73 | 1.25 | 0.7415 | 3.52 | 2.63 | 4.72 | <.0001 |
| 1-month | 2 | 1.04 | 0.79 | 1.36 | 0.7868 | 1.18 | 0.90 | 1.55 | 0.2362 | 1.18 | 0.90 | 1.56 | 0.2383 | 1.00 | 0.76 | 1.32 | 0.9947 | 1.65 | 1.24 | 2.18 | 0.0005 |
|  | 3 | 1.14 | 0.87 | 1.50 | 0.3541 | 1.28 | 0.97 | 1.68 | 0.0813 | 1.80 | 1.36 | 2.38 | <.0001 | 1.06 | 0.81 | 1.39 | 0.6518 | 2.23 | 1.67 | 2.98 | <.0001 |
|  | 4 | 1.33 | 1.01 | 1.75 | 0.043 | 1.27 | 0.96 | 1.68 | 0.0964 | 1.95 | 1.46 | 2.60 | <.0001 | 1.07 | 0.81 | 1.41 | 0.6375 | 2.32 | 1.75 | 3.08 | <.0001 |

Supplemental Table S11. Sensitivity analysis (removal of extreme pollutant estimates) of the multivariable ordinal logistic regressions for the long-term (12-months) and short-term (3-months and 1-month) associations of nitrogen dioxide (NO_2_) exposure with the 15 tested cytokines. Immune markers are reported as quartiles (reference is Quartile 1), and associations are scaled by IQR.

|  |  | **IL-1β** | | | | **IL-6** | | | | **sIL-6Rα** | | | | **IL-8** | | | | **IL-10** | | | |
| --- | --- | --- | --- | --- | --- | --- | --- | --- | --- | --- | --- | --- | --- | --- | --- | --- | --- | --- | --- | --- | --- |
|  | **Cytokine quartile** | **OR** | **L** | **U** | **P value** | **OR** | **L** | **U** | **P value** | **OR** | **L** | **U** | **P value** | **OR** | **L** | **U** | **P value** | **OR** | **L** | **U** | **P value** |
| 12-month | 2 | 1.49 | 0.90 | 2.47 | 0.1177 | 1.69 | 1.03 | 2.78 | 0.0394 | 1.13 | 0.69 | 1.86 | 0.6348 | 0.76 | 0.46 | 1.25 | 0.2853 | 0.81 | 0.50 | 1.34 | 0.4175 |
|  | 3 | 1.48 | 0.89 | 2.44 | 0.1304 | 1.46 | 0.88 | 2.42 | 0.1425 | 0.86 | 0.52 | 1.43 | 0.5577 | 0.79 | 0.48 | 1.30 | 0.3451 | 1.25 | 0.76 | 2.06 | 0.3851 |
|  | 4 | 2.30 | 1.36 | 3.87 | 0.0018 | 2.11 | 1.25 | 3.56 | 0.0054 | 1.31 | 0.79 | 2.18 | 0.2971 | 1.07 | 0.63 | 1.82 | 0.8078 | 0.87 | 0.53 | 1.45 | 0.5976 |
| 3-month | 2 | 1.75 | 1.23 | 2.50 | 0.0021 | 1.44 | 1.01 | 2.06 | 0.0445 | 1.17 | 0.82 | 1.66 | 0.3964 | 0.77 | 0.53 | 1.10 | 0.1477 | 0.79 | 0.56 | 1.13 | 0.1954 |
|  | 3 | 1.86 | 1.30 | 2.68 | 0.0007 | 1.29 | 0.90 | 1.84 | 0.1599 | 0.90 | 0.63 | 1.28 | 0.5447 | 0.89 | 0.62 | 1.28 | 0.5204 | 1.29 | 0.91 | 1.84 | 0.1572 |
|  | 4 | 2.13 | 1.48 | 3.06 | <.0001 | 1.36 | 0.95 | 1.96 | 0.0918 | 0.96 | 0.67 | 1.37 | 0.8222 | 1.37 | 0.95 | 1.96 | 0.0938 | 0.87 | 0.61 | 1.24 | 0.4484 |
| 1-month | 2 | 1.57 | 1.12 | 2.21 | 0.009 | 1.30 | 0.93 | 1.81 | 0.1245 | 1.16 | 0.84 | 1.62 | 0.3679 | 0.73 | 0.53 | 1.02 | 0.0626 | 0.82 | 0.59 | 1.14 | 0.2355 |
|  | 3 | 1.60 | 1.14 | 2.24 | 0.0065 | 1.24 | 0.89 | 1.73 | 0.2069 | 1.09 | 0.78 | 1.52 | 0.6165 | 0.70 | 0.50 | 0.99 | 0.042 | 1.25 | 0.90 | 1.72 | 0.1814 |
|  | 4 | 2.08 | 1.48 | 2.92 | <.0001 | 1.36 | 0.97 | 1.90 | 0.0752 | 1.23 | 0.88 | 1.71 | 0.2273 | 1.08 | 0.78 | 1.49 | 0.6628 | 0.99 | 0.71 | 1.37 | 0.9399 |
|  |  | **TNFα** | | | | **sTNFR2** | | | | **BAFF** | | | | **CCL2** | | | | **CCL17** | | | |
|  | **Cytokine quartile** | **OR** | **L** | **U** | **P value** | **OR** | **L** | **U** | **P value** | **OR** | **L** | **U** | **P value** | **OR** | **L** | **U** | **P value** | **OR** | **L** | **U** | **P value** |
| 12-month | 2 | 0.94 | 0.57 | 1.54 | 0.7935 | 0.88 | 0.53 | 1.44 | 0.5995 | 0.75 | 0.46 | 1.23 | 0.256 | 0.90 | 0.55 | 1.48 | 0.6907 | 0.74 | 0.45 | 1.21 | 0.2313 |
|  | 3 | 1.59 | 0.96 | 2.62 | 0.0695 | 1.22 | 0.74 | 2.03 | 0.4402 | 1.00 | 0.61 | 1.65 | 0.9967 | 0.89 | 0.54 | 1.45 | 0.6267 | 0.87 | 0.53 | 1.44 | 0.5968 |
|  | 4 | 1.37 | 0.83 | 2.28 | 0.223 | 1.46 | 0.85 | 2.50 | 0.1694 | 0.87 | 0.53 | 1.44 | 0.584 | 0.97 | 0.58 | 1.60 | 0.8916 | 0.89 | 0.55 | 1.47 | 0.6576 |
| 3-month | 2 | 1.05 | 0.73 | 1.50 | 0.7976 | 0.83 | 0.58 | 1.18 | 0.2966 | 0.90 | 0.63 | 1.28 | 0.5476 | 0.82 | 0.58 | 1.17 | 0.2807 | 0.96 | 0.67 | 1.36 | 0.8082 |
|  | 3 | 1.44 | 1.01 | 2.06 | 0.0426 | 0.89 | 0.62 | 1.28 | 0.5248 | 0.87 | 0.61 | 1.24 | 0.4396 | 0.96 | 0.68 | 1.37 | 0.8398 | 0.98 | 0.69 | 1.40 | 0.9244 |
|  | 4 | 1.46 | 1.03 | 2.07 | 0.0342 | 0.96 | 0.66 | 1.40 | 0.8425 | 0.74 | 0.52 | 1.05 | 0.0928 | 0.74 | 0.52 | 1.05 | 0.0932 | 1.41 | 0.99 | 2.02 | 0.0576 |
| 1-month | 2 | 1.11 | 0.79 | 1.55 | 0.5591 | 1.03 | 0.74 | 1.42 | 0.8848 | 1.03 | 0.74 | 1.43 | 0.8511 | 0.94 | 0.68 | 1.30 | 0.708 | 0.80 | 0.58 | 1.11 | 0.1891 |
|  | 3 | 1.65 | 1.18 | 2.29 | 0.0033 | 1.09 | 0.77 | 1.53 | 0.6314 | 1.15 | 0.83 | 1.59 | 0.4034 | 1.01 | 0.73 | 1.38 | 0.9745 | 0.85 | 0.61 | 1.19 | 0.3432 |
|  | 4 | 1.42 | 1.02 | 1.98 | 0.0374 | 1.19 | 0.84 | 1.69 | 0.336 | 0.92 | 0.66 | 1.29 | 0.6392 | 0.82 | 0.59 | 1.15 | 0.2501 | 1.04 | 0.75 | 1.44 | 0.7975 |
|  |  | **sCD14** | | | | **sCD25** | | | | **sCD27** | | | | **sCD163** | | | | **sgp130** | | | |
|  | **Cytokine quartile** | **OR** | **L** | **U** | **P value** | **OR** | **L** | **U** | **P value** | **OR** | **L** | **U** | **P value** | **OR** | **L** | **U** | **P value** | **OR** | **L** | **U** | **P value** |
| 12-month | 2 | 1.04 | 0.63 | 1.71 | 0.8771 | 0.98 | 0.60 | 1.62 | 0.9471 | 0.72 | 0.44 | 1.19 | 0.1965 | 1.11 | 0.67 | 1.83 | 0.6887 | 1.10 | 0.67 | 1.80 | 0.7086 |
|  | 3 | 0.93 | 0.56 | 1.55 | 0.7934 | 1.37 | 0.83 | 2.26 | 0.2165 | 1.20 | 0.73 | 1.98 | 0.4699 | 1.66 | 1.00 | 2.75 | 0.0504 | 0.89 | 0.54 | 1.46 | 0.6445 |
|  | 4 | 0.94 | 0.57 | 1.55 | 0.8044 | 1.29 | 0.78 | 2.15 | 0.3237 | 1.56 | 0.92 | 2.65 | 0.0955 | 1.47 | 0.89 | 2.45 | 0.1368 | 1.42 | 0.86 | 2.35 | 0.1701 |
| 3-month | 2 | 0.78 | 0.54 | 1.11 | 0.1652 | 1.11 | 0.78 | 1.58 | 0.5609 | 0.68 | 0.47 | 0.96 | 0.0288 | 1.21 | 0.85 | 1.73 | 0.2924 | 0.76 | 0.53 | 1.08 | 0.1243 |
|  | 3 | 0.78 | 0.55 | 1.12 | 0.1751 | 1.06 | 0.75 | 1.51 | 0.7319 | 0.62 | 0.43 | 0.90 | 0.011 | 1.29 | 0.91 | 1.84 | 0.1571 | 0.84 | 0.59 | 1.20 | 0.3443 |
|  | 4 | 0.77 | 0.54 | 1.10 | 0.1551 | 1.01 | 0.71 | 1.45 | 0.9488 | 1.04 | 0.72 | 1.51 | 0.823 | 1.38 | 0.96 | 1.97 | 0.0806 | 0.93 | 0.65 | 1.34 | 0.7022 |
| 1-month | 2 | 1.03 | 0.75 | 1.43 | 0.8375 | 1.44 | 1.04 | 2.00 | 0.0291 | 0.82 | 0.59 | 1.14 | 0.2277 | 1.31 | 0.94 | 1.83 | 0.1069 | 0.64 | 0.46 | 0.88 | 0.0064 |
|  | 3 | 1.07 | 0.77 | 1.49 | 0.6709 | 1.26 | 0.90 | 1.75 | 0.1802 | 0.92 | 0.66 | 1.29 | 0.6411 | 1.31 | 0.94 | 1.83 | 0.1078 | 0.68 | 0.49 | 0.94 | 0.0197 |
|  | 4 | 0.93 | 0.67 | 1.30 | 0.6621 | 1.13 | 0.81 | 1.59 | 0.4711 | 1.30 | 0.93 | 1.83 | 0.1312 | 1.27 | 0.91 | 1.78 | 0.1676 | 0.83 | 0.60 | 1.15 | 0.2526 |

Supplemental Table S12. Sensitivity analysis (removal of extreme pollutant estimates) of the multivariable ordinal logistic regressions for the long-term (12-months) and short-term (3-months and 1-month) associations of particulate matter less than 0.1µm (PM0.1) exposure with the 15 tested cytokines. Immune markers are reported as quartiles (reference is Quartile 1), and associations are scaled by IQR.

|  |  | **IL-1β** | | | | **IL-6** | | | | **sIL-6Rα** | | | | **IL-8** | | | | **IL-10** | | | |
| --- | --- | --- | --- | --- | --- | --- | --- | --- | --- | --- | --- | --- | --- | --- | --- | --- | --- | --- | --- | --- | --- |
|  | **Cytokine quartile** | **OR** | **L** | **U** | **P value** | **OR** | **L** | **U** | **P value** | **OR** | **L** | **U** | **P value** | **OR** | **L** | **U** | **P value** | **OR** | **L** | **U** | **P value** |
| 12-month | 2 | 1.06 | 0.80 | 1.39 | 0.6988 | 1.06 | 0.81 | 1.40 | 0.6541 | 1.13 | 0.85 | 1.49 | 0.3994 | 1.04 | 0.79 | 1.38 | 0.7667 | 1.12 | 0.85 | 1.48 | 0.4132 |
|  | 3 | 0.92 | 0.70 | 1.21 | 0.5480 | 0.91 | 0.69 | 1.20 | 0.4925 | 1.23 | 0.93 | 1.62 | 0.1552 | 1.03 | 0.78 | 1.36 | 0.8387 | 1.04 | 0.79 | 1.38 | 0.7697 |
|  | 4 | 0.61 | 0.46 | 0.82 | 0.0008 | 0.82 | 0.62 | 1.09 | 0.1677 | 0.99 | 0.75 | 1.31 | 0.9377 | 0.49 | 0.37 | 0.66 | <.0001 | 1.23 | 0.93 | 1.63 | 0.1520 |
| 3-month | 2 | 1.09 | 0.83 | 1.42 | 0.5446 | 1.09 | 0.83 | 1.43 | 0.5482 | 0.99 | 0.75 | 1.31 | 0.9568 | 1.47 | 1.11 | 1.95 | 0.0072 | 1.33 | 1.01 | 1.75 | 0.0446 |
|  | 3 | 0.88 | 0.67 | 1.16 | 0.3775 | 1.07 | 0.81 | 1.41 | 0.6425 | 1.16 | 0.88 | 1.53 | 0.2919 | 1.18 | 0.88 | 1.57 | 0.2684 | 1.01 | 0.75 | 1.34 | 0.9753 |
|  | 4 | 0.71 | 0.54 | 0.95 | 0.0204 | 0.98 | 0.74 | 1.31 | 0.9095 | 1.07 | 0.80 | 1.41 | 0.6606 | 0.93 | 0.69 | 1.25 | 0.6145 | 1.32 | 1.00 | 1.75 | 0.0508 |
| 1-month | 2 | 1.08 | 0.81 | 1.43 | 0.6061 | 1.00 | 0.75 | 1.32 | 0.9736 | 1.03 | 0.78 | 1.37 | 0.8381 | 1.19 | 0.89 | 1.58 | 0.2438 | 1.38 | 1.04 | 1.83 | 0.0272 |
|  | 3 | 0.97 | 0.73 | 1.29 | 0.8323 | 1.00 | 0.76 | 1.33 | 0.9779 | 1.08 | 0.81 | 1.43 | 0.6032 | 0.99 | 0.74 | 1.33 | 0.9482 | 1.03 | 0.77 | 1.37 | 0.8560 |
|  | 4 | 0.72 | 0.54 | 0.97 | 0.0305 | 0.84 | 0.63 | 1.12 | 0.2399 | 0.84 | 0.63 | 1.13 | 0.2513 | 1.02 | 0.76 | 1.36 | 0.9222 | 1.21 | 0.91 | 1.61 | 0.2005 |
|  |  | **TNFα** | | | | **sTNFR2** | | | | **BAFF** | | | | **CCL2** | | | | **CCL17** | | | |
|  | **Cytokine quartile** | **OR** | **L** | **U** | **P value** | **OR** | **L** | **U** | **P value** | **OR** | **L** | **U** | **P value** | **OR** | **L** | **U** | **P value** | **OR** | **L** | **U** | **P value** |
| 12-month | 2 | 1.07 | 0.81 | 1.41 | 0.6345 | 0.93 | 0.71 | 1.21 | 0.5736 | 1.17 | 0.88 | 1.54 | 0.2804 | 1.26 | 0.96 | 1.66 | 0.0978 | 1.01 | 0.77 | 1.34 | 0.9245 |
|  | 3 | 1.17 | 0.89 | 1.55 | 0.2581 | 0.77 | 0.58 | 1.02 | 0.0685 | 0.91 | 0.69 | 1.20 | 0.4903 | 1.14 | 0.87 | 1.50 | 0.3551 | 0.97 | 0.74 | 1.28 | 0.8286 |
|  | 4 | 0.98 | 0.74 | 1.30 | 0.8883 | 0.59 | 0.43 | 0.79 | 0.0006 | 1.07 | 0.81 | 1.42 | 0.6227 | 1.03 | 0.78 | 1.36 | 0.8291 | 1.09 | 0.83 | 1.44 | 0.5266 |
| 3-month | 2 | 1.12 | 0.85 | 1.48 | 0.4247 | 1.05 | 0.80 | 1.37 | 0.7509 | 1.54 | 1.17 | 2.04 | 0.0022 | 1.50 | 1.13 | 1.98 | 0.0053 | 1.02 | 0.78 | 1.35 | 0.8699 |
|  | 3 | 1.21 | 0.92 | 1.60 | 0.1812 | 0.82 | 0.61 | 1.09 | 0.1667 | 1.11 | 0.84 | 1.47 | 0.4776 | 1.71 | 1.29 | 2.27 | 0.0002 | 0.96 | 0.73 | 1.27 | 0.7835 |
|  | 4 | 1.08 | 0.82 | 1.43 | 0.5751 | 0.83 | 0.62 | 1.13 | 0.2354 | 1.16 | 0.87 | 1.54 | 0.3101 | 1.41 | 1.06 | 1.88 | 0.0202 | 0.93 | 0.70 | 1.23 | 0.6114 |
| 1-month | 2 | 1.11 | 0.84 | 1.47 | 0.4630 | 0.99 | 0.75 | 1.31 | 0.9409 | 1.38 | 1.04 | 1.83 | 0.0267 | 1.09 | 0.82 | 1.45 | 0.5571 | 1.35 | 1.02 | 1.79 | 0.0380 |
|  | 3 | 0.90 | 0.68 | 1.20 | 0.4736 | 0.73 | 0.54 | 0.98 | 0.0350 | 0.88 | 0.66 | 1.17 | 0.3652 | 1.33 | 1.00 | 1.76 | 0.0473 | 1.13 | 0.85 | 1.51 | 0.4001 |
|  | 4 | 0.90 | 0.68 | 1.20 | 0.4723 | 0.68 | 0.50 | 0.93 | 0.0158 | 1.03 | 0.77 | 1.37 | 0.8375 | 1.19 | 0.90 | 1.59 | 0.2260 | 1.26 | 0.95 | 1.68 | 0.1104 |
|  |  | **sCD14** | | | | **sCD25** | | | | **sCD27** | | | | **sCD163** | | | | **sgp130** | | | |
|  | **Cytokine quartile** | **OR** | **L** | **U** | **P value** | **OR** | **L** | **U** | **P value** | **OR** | **L** | **U** | **P value** | **OR** | **L** | **U** | **P value** | **OR** | **L** | **U** | **P value** |
| 12-month | 2 | 0.91 | 0.69 | 1.20 | 0.5060 | 1.00 | 0.76 | 1.31 | 0.9815 | 1.07 | 0.81 | 1.40 | 0.6445 | 0.90 | 0.68 | 1.18 | 0.4351 | 0.98 | 0.74 | 1.29 | 0.8632 |
|  | 3 | 0.69 | 0.52 | 0.92 | 0.0108 | 1.04 | 0.79 | 1.37 | 0.7919 | 0.80 | 0.60 | 1.05 | 0.1063 | 0.89 | 0.67 | 1.17 | 0.3910 | 0.86 | 0.65 | 1.13 | 0.2718 |
|  | 4 | 0.69 | 0.52 | 0.92 | 0.0103 | 0.82 | 0.62 | 1.09 | 0.1640 | 0.70 | 0.52 | 0.94 | 0.0168 | 0.89 | 0.67 | 1.18 | 0.4213 | 0.67 | 0.50 | 0.89 | 0.0052 |
| 3-month | 2 | 1.13 | 0.85 | 1.49 | 0.4002 | 1.22 | 0.92 | 1.61 | 0.1613 | 1.24 | 0.94 | 1.62 | 0.1294 | 1.05 | 0.80 | 1.38 | 0.7398 | 1.03 | 0.78 | 1.36 | 0.8298 |
|  | 3 | 1.03 | 0.78 | 1.35 | 0.8620 | 1.39 | 1.05 | 1.83 | 0.0215 | 1.16 | 0.87 | 1.53 | 0.3077 | 1.24 | 0.94 | 1.62 | 0.1296 | 0.95 | 0.72 | 1.26 | 0.7326 |
|  | 4 | 0.92 | 0.69 | 1.22 | 0.5510 | 1.04 | 0.78 | 1.39 | 0.7835 | 0.87 | 0.65 | 1.18 | 0.3671 | 0.92 | 0.69 | 1.23 | 0.5726 | 0.74 | 0.55 | 1.00 | 0.0472 |
| 1-month | 2 | 0.82 | 0.62 | 1.09 | 0.1747 | 0.96 | 0.72 | 1.27 | 0.7491 | 1.03 | 0.78 | 1.36 | 0.8533 | 0.98 | 0.74 | 1.30 | 0.8953 | 0.88 | 0.67 | 1.17 | 0.3754 |
|  | 3 | 0.67 | 0.50 | 0.89 | 0.0054 | 1.09 | 0.83 | 1.45 | 0.5319 | 0.86 | 0.65 | 1.15 | 0.3174 | 1.20 | 0.91 | 1.59 | 0.2027 | 0.78 | 0.59 | 1.04 | 0.0928 |
|  | 4 | 0.76 | 0.57 | 1.00 | 0.0535 | 0.96 | 0.72 | 1.28 | 0.7698 | 0.67 | 0.50 | 0.91 | 0.0092 | 0.90 | 0.68 | 1.21 | 0.4984 | 0.71 | 0.53 | 0.95 | 0.0196 |

Supplemental Table S13. Sensitivity analysis (removal of extreme pollutant estimates) of the multivariable ordinal logistic regressions for the long-term (12-months) and short-term (3-months and 1-month) associations of particulate matter less than 2.5µm (PM2.5) exposure with the 15 tested cytokines. Immune markers are reported as quartiles (reference is Quartile 1), and associations are scaled by IQR.

|  |  | **IL-1β** | | | | **IL-6** | | | | **sIL-6Rα** | | | | **IL-8** | | | | **IL-10** | | | |
| --- | --- | --- | --- | --- | --- | --- | --- | --- | --- | --- | --- | --- | --- | --- | --- | --- | --- | --- | --- | --- | --- |
|  | **Cytokine quartile** | **OR** | **L** | **U** | **P value** | **OR** | **L** | **U** | **P value** | **OR** | **L** | **U** | **P value** | **OR** | **L** | **U** | **P value** | **OR** | **L** | **U** | **P value** |
| 12-month | 2 | 1.20 | 0.80 | 1.79 | 0.388 | 0.83 | 0.56 | 1.25 | 0.3806 | 0.69 | 0.46 | 1.04 | 0.074 | 1.09 | 0.72 | 1.63 | 0.6907 | 1.24 | 0.83 | 1.86 | 0.2978 |
|  | 3 | 1.13 | 0.75 | 1.70 | 0.5694 | 0.92 | 0.61 | 1.39 | 0.6849 | 1.07 | 0.71 | 1.61 | 0.7659 | 1.26 | 0.84 | 1.89 | 0.2582 | 0.87 | 0.58 | 1.31 | 0.5077 |
|  | 4 | 0.78 | 0.51 | 1.19 | 0.2429 | 0.63 | 0.41 | 0.96 | 0.0312 | 0.73 | 0.49 | 1.11 | 0.1377 | 0.86 | 0.57 | 1.31 | 0.4943 | 1.28 | 0.85 | 1.93 | 0.2399 |
| 3-month | 2 | 0.89 | 0.65 | 1.22 | 0.4529 | 0.96 | 0.70 | 1.32 | 0.7907 | 0.85 | 0.61 | 1.16 | 0.2987 | 1.13 | 0.82 | 1.57 | 0.4461 | 1.25 | 0.91 | 1.71 | 0.166 |
|  | 3 | 0.80 | 0.58 | 1.10 | 0.1724 | 0.97 | 0.70 | 1.33 | 0.845 | 1.19 | 0.86 | 1.63 | 0.2931 | 1.11 | 0.80 | 1.54 | 0.5237 | 0.89 | 0.65 | 1.22 | 0.466 |
|  | 4 | 0.92 | 0.67 | 1.27 | 0.6104 | 1.03 | 0.75 | 1.43 | 0.8482 | 1.02 | 0.74 | 1.41 | 0.8856 | 1.15 | 0.83 | 1.59 | 0.3901 | 1.21 | 0.88 | 1.66 | 0.2433 |
| 1-month | 2 | 1.03 | 0.77 | 1.39 | 0.8304 | 0.90 | 0.67 | 1.22 | 0.5084 | 0.72 | 0.53 | 0.97 | 0.0325 | 1.28 | 0.95 | 1.73 | 0.1057 | 1.12 | 0.83 | 1.51 | 0.456 |
|  | 3 | 0.87 | 0.64 | 1.18 | 0.3715 | 0.99 | 0.73 | 1.34 | 0.9595 | 1.06 | 0.79 | 1.43 | 0.7082 | 1.53 | 1.12 | 2.08 | 0.0071 | 0.87 | 0.65 | 1.17 | 0.3492 |
|  | 4 | 0.85 | 0.63 | 1.15 | 0.2792 | 0.89 | 0.66 | 1.21 | 0.4609 | 0.72 | 0.53 | 0.98 | 0.0377 | 1.26 | 0.93 | 1.70 | 0.1385 | 1.02 | 0.76 | 1.38 | 0.8929 |
|  |  | **TNFα** | | | | **sTNFR2** | | | | **BAFF** | | | | **CCL2** | | | | **CCL17** | | | |
|  | **Cytokine quartile** | **OR** | **L** | **U** | **P value** | **OR** | **L** | **U** | **P value** | **OR** | **L** | **U** | **P value** | **OR** | **L** | **U** | **P value** | **OR** | **L** | **U** | **P value** |
| 12-month | 2 | 1.15 | 0.77 | 1.72 | 0.5067 | 1.00 | 0.66 | 1.50 | 0.9918 | 1.25 | 0.83 | 1.88 | 0.287 | 1.59 | 1.05 | 2.40 | 0.0269 | 1.06 | 0.71 | 1.60 | 0.77 |
|  | 3 | 0.82 | 0.54 | 1.23 | 0.3291 | 0.97 | 0.64 | 1.47 | 0.8986 | 1.01 | 0.67 | 1.52 | 0.9804 | 1.28 | 0.85 | 1.92 | 0.2434 | 1.11 | 0.74 | 1.67 | 0.6187 |
|  | 4 | 0.89 | 0.59 | 1.34 | 0.5785 | 0.74 | 0.48 | 1.15 | 0.1836 | 0.96 | 0.64 | 1.46 | 0.8636 | 1.32 | 0.87 | 1.99 | 0.1924 | 1.11 | 0.74 | 1.66 | 0.62 |
| 3-month | 2 | 1.14 | 0.83 | 1.57 | 0.4045 | 1.14 | 0.83 | 1.58 | 0.4178 | 1.30 | 0.94 | 1.80 | 0.1072 | 1.48 | 1.08 | 2.04 | 0.0162 | 1.07 | 0.78 | 1.46 | 0.6985 |
|  | 3 | 1.00 | 0.73 | 1.38 | 0.9792 | 1.12 | 0.81 | 1.55 | 0.5083 | 1.38 | 1.01 | 1.90 | 0.0464 | 1.13 | 0.82 | 1.55 | 0.4638 | 1.12 | 0.82 | 1.54 | 0.4736 |
|  | 4 | 0.93 | 0.68 | 1.28 | 0.6713 | 1.05 | 0.74 | 1.47 | 0.8012 | 1.23 | 0.89 | 1.70 | 0.2072 | 1.59 | 1.15 | 2.19 | 0.0046 | 0.89 | 0.65 | 1.23 | 0.4842 |
| 1-month | 2 | 1.06 | 0.79 | 1.44 | 0.6877 | 1.04 | 0.77 | 1.40 | 0.7857 | 1.04 | 0.78 | 1.41 | 0.7755 | 1.09 | 0.81 | 1.47 | 0.5626 | 1.42 | 1.05 | 1.92 | 0.0214 |
|  | 3 | 0.97 | 0.72 | 1.31 | 0.824 | 0.93 | 0.69 | 1.27 | 0.6553 | 0.89 | 0.66 | 1.20 | 0.4398 | 1.10 | 0.82 | 1.48 | 0.5337 | 1.33 | 0.98 | 1.79 | 0.0681 |
|  | 4 | 0.92 | 0.68 | 1.25 | 0.602 | 0.90 | 0.65 | 1.24 | 0.5168 | 0.90 | 0.66 | 1.21 | 0.4723 | 1.37 | 1.01 | 1.85 | 0.0422 | 1.19 | 0.88 | 1.60 | 0.2592 |
|  |  | **sCD14** | | | | **sCD25** | | | | **sCD27** | | | | **sCD163** | | | | **sgp130** | | | |
|  | **Cytokine quartile** | **OR** | **L** | **U** | **P value** | **OR** | **L** | **U** | **P value** | **OR** | **L** | **U** | **P value** | **OR** | **L** | **U** | **P value** | **OR** | **L** | **U** | **P value** |
| 12-month | 2 | 0.92 | 0.61 | 1.39 | 0.7062 | 1.13 | 0.75 | 1.70 | 0.5513 | 1.01 | 0.67 | 1.52 | 0.9498 | 0.92 | 0.61 | 1.39 | 0.6992 | 0.84 | 0.56 | 1.26 | 0.3985 |
|  | 3 | 0.93 | 0.62 | 1.41 | 0.7387 | 0.82 | 0.54 | 1.23 | 0.3262 | 0.81 | 0.54 | 1.22 | 0.3031 | 0.61 | 0.41 | 0.92 | 0.0187 | 0.74 | 0.49 | 1.11 | 0.1434 |
|  | 4 | 1.07 | 0.71 | 1.61 | 0.7581 | 0.87 | 0.58 | 1.33 | 0.528 | 0.82 | 0.54 | 1.25 | 0.3547 | 0.71 | 0.47 | 1.08 | 0.1048 | 0.77 | 0.51 | 1.16 | 0.2046 |
| 3-month | 2 | 1.32 | 0.96 | 1.82 | 0.0929 | 1.12 | 0.81 | 1.54 | 0.4937 | 1.24 | 0.90 | 1.71 | 0.1882 | 0.92 | 0.67 | 1.26 | 0.5845 | 0.92 | 0.66 | 1.27 | 0.6017 |
|  | 3 | 1.38 | 1.00 | 1.89 | 0.0509 | 1.15 | 0.84 | 1.58 | 0.387 | 1.42 | 1.03 | 1.97 | 0.0327 | 0.97 | 0.70 | 1.33 | 0.8281 | 0.92 | 0.67 | 1.28 | 0.6292 |
|  | 4 | 1.23 | 0.89 | 1.69 | 0.2147 | 1.14 | 0.82 | 1.57 | 0.4313 | 1.01 | 0.72 | 1.42 | 0.9438 | 0.87 | 0.63 | 1.19 | 0.3787 | 0.80 | 0.57 | 1.11 | 0.1822 |
| 1-month | 2 | 0.96 | 0.71 | 1.29 | 0.78 | 0.84 | 0.62 | 1.13 | 0.2352 | 0.89 | 0.66 | 1.19 | 0.4305 | 0.77 | 0.57 | 1.04 | 0.0925 | 0.98 | 0.72 | 1.31 | 0.866 |
|  | 3 | 0.96 | 0.71 | 1.29 | 0.7662 | 0.85 | 0.63 | 1.15 | 0.2791 | 0.81 | 0.60 | 1.10 | 0.1716 | 0.83 | 0.62 | 1.12 | 0.2304 | 0.90 | 0.67 | 1.22 | 0.5012 |
|  | 4 | 1.10 | 0.82 | 1.49 | 0.5198 | 0.86 | 0.64 | 1.18 | 0.3523 | 0.75 | 0.55 | 1.02 | 0.0678 | 0.82 | 0.60 | 1.11 | 0.2006 | 0.97 | 0.72 | 1.30 | 0.8199 |

Supplemental Table S14. Sensitivity analysis (removal of extreme pollutant estimates) of the multivariable ordinal logistic regressions for the long-term (12-months) and short-term (3-months and 1-month) associations of particulate matter less than 10µm (PM10) exposure with the 15 tested cytokines. Immune markers are reported as quartiles (reference is Quartile 1), and associations are scaled by IQR.

|  |  | **IL-1β** | | | | **IL-6** | | | | **sIL-6Rα** | | | | **IL-8** | | | | **IL-10** | | | |
| --- | --- | --- | --- | --- | --- | --- | --- | --- | --- | --- | --- | --- | --- | --- | --- | --- | --- | --- | --- | --- | --- |
|  | **Cytokine quartile** | **OR** | **L** | **U** | **P value** | **OR** | **L** | **U** | **P value** | **OR** | **L** | **U** | **P value** | **OR** | **L** | **U** | **P value** | **OR** | **L** | **U** | **P value** |
| 12-month | 2 | 0.83 | 0.58 | 1.18 | 0.2936 | 0.87 | 0.61 | 1.24 | 0.4404 | 0.86 | 0.61 | 1.21 | 0.3886 | 0.92 | 0.65 | 1.31 | 0.6429 | 1.13 | 0.79 | 1.60 | 0.505 |
|  | 3 | 0.94 | 0.66 | 1.35 | 0.7496 | 0.91 | 0.64 | 1.30 | 0.6072 | 0.98 | 0.69 | 1.39 | 0.9089 | 1.06 | 0.75 | 1.52 | 0.7306 | 0.88 | 0.62 | 1.26 | 0.4859 |
|  | 4 | 0.55 | 0.38 | 0.79 | 0.0013 | 0.54 | 0.37 | 0.78 | 0.001 | 0.77 | 0.54 | 1.10 | 0.1466 | 0.49 | 0.34 | 0.71 | 0.0001 | 0.99 | 0.70 | 1.41 | 0.974 |
| 3-month | 2 | 0.79 | 0.58 | 1.09 | 0.1508 | 0.82 | 0.60 | 1.13 | 0.2252 | 0.84 | 0.61 | 1.15 | 0.2758 | 1.07 | 0.77 | 1.48 | 0.692 | 1.36 | 0.99 | 1.87 | 0.0557 |
|  | 3 | 0.71 | 0.52 | 0.98 | 0.0398 | 0.95 | 0.69 | 1.30 | 0.7278 | 1.18 | 0.86 | 1.63 | 0.3008 | 0.98 | 0.71 | 1.36 | 0.9063 | 0.94 | 0.68 | 1.29 | 0.6828 |
|  | 4 | 0.69 | 0.50 | 0.95 | 0.0248 | 0.90 | 0.65 | 1.25 | 0.5393 | 1.02 | 0.74 | 1.41 | 0.912 | 0.80 | 0.57 | 1.10 | 0.1718 | 1.21 | 0.88 | 1.67 | 0.251 |
| 1-month | 2 | 0.85 | 0.62 | 1.15 | 0.2888 | 0.90 | 0.66 | 1.23 | 0.5216 | 0.78 | 0.57 | 1.06 | 0.1109 | 1.16 | 0.85 | 1.58 | 0.3489 | 1.07 | 0.79 | 1.45 | 0.6836 |
|  | 3 | 0.82 | 0.60 | 1.12 | 0.2204 | 0.89 | 0.65 | 1.22 | 0.4651 | 0.98 | 0.72 | 1.34 | 0.912 | 1.36 | 0.99 | 1.87 | 0.055 | 0.82 | 0.60 | 1.12 | 0.2053 |
|  | 4 | 0.61 | 0.45 | 0.84 | 0.0025 | 0.82 | 0.60 | 1.13 | 0.233 | 0.72 | 0.52 | 0.98 | 0.0373 | 0.82 | 0.60 | 1.13 | 0.2261 | 1.00 | 0.73 | 1.36 | 0.9962 |
|  |  | **TNFα** | | | | **sTNFR2** | | | | **BAFF** | | | | **CCL2** | | | | **CCL17** | | | |
|  | **Cytokine quartile** | **OR** | **L** | **U** | **P value** | **OR** | **L** | **U** | **P value** | **OR** | **L** | **U** | **P value** | **OR** | **L** | **U** | **P value** | **OR** | **L** | **U** | **P value** |
| 12-month | 2 | 1.09 | 0.77 | 1.55 | 0.6359 | 0.93 | 0.65 | 1.33 | 0.6797 | 1.33 | 0.94 | 1.89 | 0.1104 | 1.34 | 0.94 | 1.90 | 0.1054 | 0.91 | 0.64 | 1.29 | 0.5782 |
|  | 3 | 0.88 | 0.62 | 1.25 | 0.4688 | 1.03 | 0.72 | 1.46 | 0.8866 | 1.15 | 0.81 | 1.63 | 0.4464 | 1.20 | 0.84 | 1.71 | 0.3107 | 0.83 | 0.58 | 1.17 | 0.2825 |
|  | 4 | 0.73 | 0.51 | 1.05 | 0.0865 | 0.75 | 0.51 | 1.09 | 0.1285 | 0.88 | 0.62 | 1.27 | 0.4992 | 1.08 | 0.75 | 1.53 | 0.6897 | 0.92 | 0.65 | 1.30 | 0.6509 |
| 3-month | 2 | 1.11 | 0.81 | 1.53 | 0.5172 | 1.19 | 0.86 | 1.65 | 0.2896 | 1.16 | 0.84 | 1.60 | 0.3782 | 1.35 | 0.97 | 1.86 | 0.0735 | 1.23 | 0.89 | 1.70 | 0.2021 |
|  | 3 | 0.96 | 0.69 | 1.32 | 0.781 | 1.16 | 0.83 | 1.62 | 0.3752 | 1.25 | 0.90 | 1.72 | 0.183 | 1.17 | 0.85 | 1.62 | 0.3303 | 1.14 | 0.83 | 1.58 | 0.4119 |
|  | 4 | 0.91 | 0.67 | 1.26 | 0.582 | 1.08 | 0.76 | 1.52 | 0.6804 | 1.32 | 0.96 | 1.83 | 0.0892 | 1.57 | 1.14 | 2.17 | 0.0058 | 1.01 | 0.73 | 1.39 | 0.9641 |
| 1-month | 2 | 1.04 | 0.77 | 1.43 | 0.7847 | 0.94 | 0.69 | 1.28 | 0.6854 | 0.94 | 0.69 | 1.27 | 0.6812 | 1.11 | 0.81 | 1.51 | 0.5142 | 1.48 | 1.08 | 2.02 | 0.0147 |
|  | 3 | 0.91 | 0.66 | 1.24 | 0.5378 | 0.89 | 0.65 | 1.23 | 0.4773 | 0.80 | 0.59 | 1.09 | 0.1592 | 1.00 | 0.73 | 1.35 | 0.9823 | 1.23 | 0.90 | 1.69 | 0.1912 |
|  | 4 | 0.84 | 0.62 | 1.15 | 0.2816 | 0.84 | 0.60 | 1.17 | 0.3086 | 0.88 | 0.64 | 1.20 | 0.4024 | 1.18 | 0.86 | 1.62 | 0.2962 | 1.17 | 0.85 | 1.60 | 0.3345 |
|  |  | **sCD14** | | | | **sCD25** | | | | **sCD27** | | | | **sCD163** | | | | **sgp130** | | | |
|  | **Cytokine quartile** | **OR** | **L** | **U** | **P value** | **OR** | **L** | **U** | **P value** | **OR** | **L** | **U** | **P value** | **OR** | **L** | **U** | **P value** | **OR** | **L** | **U** | **P value** |
| 12-month | 2 | 0.99 | 0.69 | 1.40 | 0.931 | 1.11 | 0.78 | 1.57 | 0.5689 | 1.10 | 0.77 | 1.56 | 0.6052 | 0.93 | 0.65 | 1.32 | 0.6812 | 0.86 | 0.61 | 1.22 | 0.4084 |
|  | 3 | 1.00 | 0.70 | 1.43 | 0.9885 | 0.84 | 0.59 | 1.20 | 0.3389 | 1.03 | 0.73 | 1.46 | 0.8659 | 0.65 | 0.46 | 0.93 | 0.0193 | 0.86 | 0.61 | 1.21 | 0.383 |
|  | 4 | 1.28 | 0.91 | 1.82 | 0.1627 | 0.93 | 0.65 | 1.32 | 0.6698 | 0.85 | 0.59 | 1.23 | 0.391 | 0.83 | 0.58 | 1.18 | 0.2959 | 0.87 | 0.62 | 1.24 | 0.4478 |
| 3-month | 2 | 1.29 | 0.93 | 1.79 | 0.1305 | 1.07 | 0.77 | 1.47 | 0.6964 | 1.15 | 0.84 | 1.59 | 0.3913 | 0.96 | 0.70 | 1.32 | 0.8063 | 0.91 | 0.66 | 1.26 | 0.5634 |
|  | 3 | 1.38 | 0.99 | 1.91 | 0.054 | 1.17 | 0.85 | 1.61 | 0.3285 | 1.28 | 0.92 | 1.77 | 0.1393 | 0.93 | 0.67 | 1.28 | 0.6411 | 0.91 | 0.66 | 1.27 | 0.5867 |
|  | 4 | 1.53 | 1.10 | 2.12 | 0.0106 | 1.12 | 0.81 | 1.56 | 0.4835 | 0.90 | 0.64 | 1.26 | 0.5212 | 0.97 | 0.70 | 1.34 | 0.839 | 0.85 | 0.61 | 1.18 | 0.3275 |
| 1-month | 2 | 0.98 | 0.72 | 1.34 | 0.9076 | 0.84 | 0.61 | 1.13 | 0.2482 | 0.82 | 0.61 | 1.12 | 0.2128 | 0.83 | 0.61 | 1.13 | 0.2382 | 1.01 | 0.74 | 1.37 | 0.9743 |
|  | 3 | 1.03 | 0.75 | 1.40 | 0.8636 | 0.84 | 0.61 | 1.14 | 0.2567 | 0.83 | 0.61 | 1.13 | 0.2302 | 0.84 | 0.61 | 1.14 | 0.2621 | 0.94 | 0.69 | 1.28 | 0.6817 |
|  | 4 | 1.17 | 0.86 | 1.60 | 0.3282 | 0.85 | 0.62 | 1.17 | 0.31 | 0.72 | 0.53 | 0.99 | 0.0453 | 0.95 | 0.70 | 1.30 | 0.7584 | 1.00 | 0.73 | 1.36 | 0.9801 |

Supplemental Table S15. Multivariable linear regressions for the long-term (12-months) and short-term (3-months and 1-month) associations of pollutants with the 15 tested immune markers (continuous variables; per pg/ml).

|  |  | **IL-1β** | | | | **IL-6** | | | | **sIL-6Rα** | | | | **IL-8** | | | |
| --- | --- | --- | --- | --- | --- | --- | --- | --- | --- | --- | --- | --- | --- | --- | --- | --- | --- |
|  | **Time** | **Est** | **SE** | **P** | **95% CI** | **Est** | **SE** | **P** | **95% CI** | **Est** | **SE** | **P** | **95% CI** | **Est** | **SE** | **P** | **95% CI** |
| Ozone | 12-month | 0.286 | 0.034 | <.0001 | 0.219-0.354 | 0.065 | 0.029 | 0.0236 | 0.009-0.121 | -0.004 | 0.009 | 0.6271 | -0.022-0.013 | 0.522 | 0.039 | <.0001 | 0.445-0.599 |
|  | 3-month | 0.162 | 0.035 | <.0001 | 0.094-0.23 | 0.028 | 0.029 | 0.3293 | -0.028-0.084 | -0.002 | 0.009 | 0.8645 | -0.019-0.016 | 0.287 | 0.041 | <.0001 | 0.207-0.367 |
|  | 1-month | 0.144 | 0.033 | <.0001 | 0.08-0.209 | 0.011 | 0.027 | 0.6973 | -0.042-0.063 | -0.001 | 0.008 | 0.9288 | -0.017-0.016 | 0.248 | 0.039 | <.0001 | 0.172-0.324 |
| NO2 | 12-month | 0.197 | 0.068 | 0.0037 | 0.064-0.33 | 0.063 | 0.056 | 0.2634 | -0.048-0.174 | -0.015 | 0.018 | 0.3954 | -0.05-0.02 | 0.294 | 0.077 | 0.0001 | 0.143-0.446 |
|  | 3-month | 0.152 | 0.044 | 0.0006 | 0.066-0.239 | 0.053 | 0.036 | 0.146 | -0.018-0.124 | -0.006 | 0.011 | 0.6201 | -0.028-0.017 | 0.116 | 0.052 | 0.0248 | 0.015-0.217 |
|  | 1-month | 0.119 | 0.041 | 0.0043 | 0.037-0.2 | 0.055 | 0.034 | 0.1043 | -0.011-0.122 | 0.008 | 0.011 | 0.4678 | -0.013-0.028 | 0.033 | 0.049 | 0.4963 | -0.062-0.128 |
| PM0.1 | 12-month | -0.114 | 0.034 | 0.0008 | -0.181--0.048 | -0.047 | 0.028 | 0.0928 | -0.102-0.008 | 0.003 | 0.009 | 0.7261 | -0.014-0.02 | -0.310 | 0.040 | <.0001 | -0.387--0.232 |
|  | 3-month | -0.087 | 0.033 | 0.0091 | -0.153--0.022 | -0.008 | 0.027 | 0.7629 | -0.062-0.045 | 0.002 | 0.009 | 0.8388 | -0.015-0.018 | -0.155 | 0.040 | <.0001 | -0.233--0.077 |
|  | 1-month | -0.056 | 0.029 | 0.0551 | -0.114-0.001 | -0.025 | 0.024 | 0.3016 | -0.072-0.022 | -0.009 | 0.007 | 0.2316 | -0.024-0.006 | -0.071 | 0.035 | 0.0391 | -0.139--0.004 |
| PM2.5 | 12-month | -0.075 | 0.060 | 0.2116 | -0.193-0.043 | -0.008 | 0.050 | 0.8691 | -0.105-0.089 | 0.003 | 0.015 | 0.8457 | -0.027-0.033 | -0.169 | 0.071 | 0.0181 | -0.308--0.029 |
|  | 3-month | -0.031 | 0.032 | 0.3345 | -0.094-0.032 | -0.023 | 0.026 | 0.3733 | -0.075-0.028 | -0.002 | 0.008 | 0.8492 | -0.018-0.014 | -0.034 | 0.038 | 0.3679 | -0.109-0.04 |
|  | 1-month | -0.049 | 0.029 | 0.0972 | -0.107-0.009 | -0.038 | 0.024 | 0.1119 | -0.086-0.009 | -0.016 | 0.008 | 0.0355 | -0.031--0.001 | -0.044 | 0.035 | 0.209 | -0.112-0.025 |
| PM10 | 12-month | -0.225 | 0.046 | <.0001 | -0.314--0.135 | -0.055 | 0.038 | 0.1456 | -0.129-0.019 | 0.011 | 0.012 | 0.3674 | -0.013-0.034 | -0.440 | 0.054 | <.0001 | -0.545--0.335 |
|  | 3-month | -0.068 | 0.025 | 0.0073 | -0.118--0.018 | -0.035 | 0.021 | 0.0954 | -0.075-0.006 | 0.003 | 0.006 | 0.6335 | -0.01-0.016 | -0.126 | 0.030 | <.0001 | -0.185--0.067 |
|  | 1-month | -0.076 | 0.026 | 0.003 | -0.126--0.026 | -0.043 | 0.021 | 0.0414 | -0.084--0.002 | -0.006 | 0.007 | 0.3419 | -0.019-0.007 | -0.108 | 0.030 | 0.0003 | -0.167--0.049 |
|  |  | **IL-10** | | | | **TNFα** | | | | **sTNFR2** | | | | **BAFF** | | | |
|  | **Time** | **Est** | **SE** | **P** | **95% CI** | **Est** | **SE** | **P** | **95% CI** | **Est** | **SE** | **P** | **95% CI** | **Est** | **SE** | **P** | **95% CI** |
| Ozone | 12-month | 0.042 | 0.028 | 0.1315 | -0.013-0.097 | 0.039 | 0.017 | 0.0192 | 0.006-0.071 | 0.072 | 0.014 | <.0001 | 0.044-0.099 | 0.014 | 0.008 | 0.1068 | -0.003-0.03 |
|  | 3-month | 0.020 | 0.028 | 0.4691 | -0.035-0.075 | 0.021 | 0.016 | 0.1968 | -0.011-0.054 | 0.081 | 0.014 | <.0001 | 0.054-0.108 | 0.025 | 0.008 | 0.0028 | 0.009-0.041 |
|  | 1-month | 0.028 | 0.026 | 0.2893 | -0.024-0.08 | 0.017 | 0.016 | 0.2701 | -0.013-0.048 | 0.072 | 0.013 | <.0001 | 0.047-0.098 | 0.023 | 0.008 | 0.0036 | 0.008-0.038 |
| NO2 | 12-month | 0.029 | 0.055 | 0.6043 | -0.08-0.137 | 0.026 | 0.033 | 0.4214 | -0.038-0.09 | 0.051 | 0.027 | 0.061 | -0.002-0.105 | -0.003 | 0.017 | 0.8599 | -0.035-0.029 |
|  | 3-month | 0.075 | 0.035 | 0.0355 | 0.005-0.144 | 0.053 | 0.021 | 0.0112 | 0.012-0.094 | 0.026 | 0.017 | 0.1413 | -0.009-0.06 | -0.003 | 0.011 | 0.8037 | -0.023-0.018 |
|  | 1-month | 0.073 | 0.033 | 0.029 | 0.007-0.138 | 0.044 | 0.020 | 0.0234 | 0.006-0.083 | 0.032 | 0.016 | 0.0521 | 0-0.064 | 0.004 | 0.010 | 0.6708 | -0.015-0.024 |
| PM0.1 | 12-month | -0.006 | 0.027 | 0.819 | -0.06-0.047 | -0.009 | 0.016 | 0.5928 | -0.04-0.023 | -0.048 | 0.013 | 0.0004 | -0.074--0.021 | -0.004 | 0.008 | 0.6165 | -0.02-0.012 |
|  | 3-month | -0.015 | 0.027 | 0.5641 | -0.068-0.037 | -0.008 | 0.016 | 0.5989 | -0.039-0.023 | -0.033 | 0.013 | 0.0132 | -0.059--0.007 | -0.003 | 0.008 | 0.7115 | -0.019-0.013 |
|  | 1-month | -0.029 | 0.023 | 0.2241 | -0.075-0.017 | -0.019 | 0.014 | 0.16 | -0.047-0.008 | -0.038 | 0.012 | 0.0011 | -0.06--0.015 | -0.007 | 0.007 | 0.3395 | -0.02-0.007 |
| PM2.5 | 12-month | 0.043 | 0.049 | 0.3704 | -0.052-0.139 | 0.015 | 0.029 | 0.6049 | -0.041-0.071 | -0.020 | 0.024 | 0.3987 | -0.067-0.027 | 0.001 | 0.014 | 0.9361 | -0.027-0.03 |
|  | 3-month | -0.028 | 0.026 | 0.2752 | -0.078-0.022 | -0.025 | 0.015 | 0.1028 | -0.054-0.005 | -0.005 | 0.013 | 0.7013 | -0.03-0.02 | 0.002 | 0.008 | 0.7791 | -0.013-0.017 |
|  | 1-month | -0.027 | 0.024 | 0.2503 | -0.073-0.019 | -0.023 | 0.014 | 0.0915 | -0.051-0.004 | -0.019 | 0.012 | 0.1048 | -0.042-0.004 | -0.010 | 0.007 | 0.1697 | -0.023-0.004 |
| PM10 | 12-month | -0.027 | 0.037 | 0.4664 | -0.1-0.046 | -0.016 | 0.022 | 0.4707 | -0.059-0.027 | -0.028 | 0.018 | 0.1233 | -0.064-0.008 | 0.003 | 0.011 | 0.7623 | -0.018-0.025 |
|  | 3-month | -0.031 | 0.020 | 0.1264 | -0.071-0.009 | -0.023 | 0.012 | 0.0522 | -0.047-0 | -0.007 | 0.010 | 0.4658 | -0.027-0.012 | 0.002 | 0.006 | 0.7242 | -0.01-0.014 |
|  | 1-month | -0.033 | 0.020 | 0.108 | -0.073-0.007 | -0.024 | 0.012 | 0.046 | -0.048-0 | -0.012 | 0.010 | 0.2276 | -0.032-0.008 | -0.004 | 0.006 | 0.5624 | -0.015-0.008 |
|  |  | **CCL2** | | | | **CCL17** | | | | **sCD14** | | | | **sCD25** | | | |
|  | **Time** | **Est** | **SE** | **P** | **95% CI** | **Est** | **SE** | **P** | **95% CI** | **Est** | **SE** | **P** | **95% CI** | **Est** | **SE** | **P** | **95% CI** |
| Ozone | 12-month | 0.042 | 0.030 | 0.1642 | -0.017-0.102 | -0.040 | 0.038 | 0.2957 | -0.115-0.035 | 0.001 | 0.006 | 0.9275 | -0.012-0.013 | 0.025 | 0.012 | 0.049 | 0-0.049 |
|  | 3-month | -0.030 | 0.030 | 0.3231 | -0.089-0.029 | 0.001 | 0.038 | 0.9859 | -0.074-0.075 | 0.014 | 0.006 | 0.0202 | 0.002-0.026 | 0.021 | 0.012 | 0.0912 | -0.003-0.045 |
|  | 1-month | -0.015 | 0.029 | 0.6006 | -0.071-0.041 | -0.006 | 0.036 | 0.8593 | -0.077-0.064 | 0.014 | 0.006 | 0.0164 | 0.003-0.025 | 0.016 | 0.012 | 0.1712 | -0.007-0.039 |
| NO2 | 12-month | 0.011 | 0.060 | 0.8488 | -0.106-0.129 | 0.058 | 0.075 | 0.4405 | -0.089-0.205 | -0.008 | 0.012 | 0.4986 | -0.032-0.016 | 0.037 | 0.024 | 0.1309 | -0.011-0.085 |
|  | 3-month | 0.007 | 0.038 | 0.8479 | -0.068-0.083 | 0.043 | 0.048 | 0.3685 | -0.051-0.137 | -0.006 | 0.008 | 0.4654 | -0.021-0.01 | 0.033 | 0.016 | 0.0359 | 0.002-0.064 |
|  | 1-month | 0.004 | 0.036 | 0.9083 | -0.066-0.075 | 0.005 | 0.045 | 0.9089 | -0.083-0.093 | -0.007 | 0.007 | 0.363 | -0.021-0.008 | 0.032 | 0.015 | 0.0298 | 0.003-0.061 |
| PM0.1 | 12-month | -0.016 | 0.030 | 0.5943 | -0.074-0.042 | 0.002 | 0.037 | 0.9487 | -0.07-0.075 | -0.011 | 0.006 | 0.0632 | -0.023-0.001 | -0.022 | 0.012 | 0.066 | -0.046-0.001 |
|  | 3-month | 0.049 | 0.029 | 0.0923 | -0.008-0.106 | -0.047 | 0.036 | 0.1968 | -0.118-0.024 | -0.005 | 0.006 | 0.4314 | -0.016-0.007 | -0.015 | 0.012 | 0.2024 | -0.038-0.008 |
|  | 1-month | 0.034 | 0.025 | 0.1797 | -0.016-0.084 | 0.000 | 0.032 | 0.9887 | -0.063-0.062 | -0.008 | 0.005 | 0.1339 | -0.018-0.002 | -0.013 | 0.010 | 0.2056 | -0.034-0.007 |
|  |  | **CCL2** | | | | **CCL17** | | | | **sCD14** | | | | **sCD25** | | | |
|  | **Time** | **Est** | **SE** | **P** | **95% CI** | **Est** | **SE** | **P** | **95% CI** | **Est** | **SE** | **P** | **95% CI** | **Est** | **SE** | **P** | **95% CI** |
| PM2.5 | 12-month | 0.041 | 0.053 | 0.4333 | -0.062-0.144 | -0.067 | 0.066 | 0.3072 | -0.196-0.062 | 0.008 | 0.011 | 0.477 | -0.013-0.029 | -0.027 | 0.021 | 0.21 | -0.069-0.015 |
|  | 3-month | 0.012 | 0.028 | 0.6638 | -0.042-0.067 | -0.060 | 0.035 | 0.0849 | -0.128-0.008 | -0.005 | 0.006 | 0.4183 | -0.016-0.006 | -0.021 | 0.011 | 0.0579 | -0.044-0.001 |
|  | 1-month | 0.013 | 0.026 | 0.5997 | -0.037-0.064 | -0.057 | 0.032 | 0.076 | -0.119-0.006 | -0.004 | 0.005 | 0.4202 | -0.014-0.006 | -0.026 | 0.010 | 0.0125 | -0.047--0.006 |
| PM10 | 12-month | 0.011 | 0.040 | 0.7772 | -0.067-0.09 | -0.043 | 0.050 | 0.3958 | -0.141-0.056 | 0.011 | 0.008 | 0.1721 | -0.005-0.027 | -0.017 | 0.016 | 0.2938 | -0.049-0.015 |
|  | 3-month | -0.003 | 0.022 | 0.8966 | -0.046-0.04 | -0.038 | 0.028 | 0.1643 | -0.092-0.016 | -0.001 | 0.004 | 0.7754 | -0.01-0.007 | -0.007 | 0.009 | 0.422 | -0.025-0.01 |
|  | 1-month | -0.017 | 0.022 | 0.4407 | -0.061-0.026 | -0.037 | 0.028 | 0.1811 | -0.091-0.017 | 0.001 | 0.004 | 0.8175 | -0.008-0.01 | -0.011 | 0.009 | 0.2218 | -0.029-0.007 |
|  |  | **sCD27** | | | | **sCD163** | | | | **sgp130** | | | |  |  |  |  |
|  | **Time** | **Est** | **SE** | **P** | **95% CI** | **Est** | **SE** | **P** | **95% CI** | **Est** | **SE** | **P** | **95% CI** |  |  |  |  |
| Ozone | 12-month | 0.019 | 0.012 | 0.1095 | -0.004-0.042 | 0.070 | 0.035 | 0.0491 | 0-0.139 | 0.015 | 0.008 | 0.0558 | 0-0.03 |  |  |  |  |
|  | 3-month | 0.052 | 0.012 | <.0001 | 0.029-0.075 | 0.027 | 0.035 | 0.4398 | -0.042-0.096 | 0.073 | 0.008 | <.0001 | 0.058-0.088 |  |  |  |  |
|  | 1-month | 0.046 | 0.011 | <.0001 | 0.024-0.067 | 0.002 | 0.033 | 0.9624 | -0.064-0.067 | 0.050 | 0.007 | <.0001 | 0.036-0.065 |  |  |  |  |
| NO2 | 12-month | 0.051 | 0.023 | 0.0278 | 0.006-0.096 | -0.062 | 0.070 | 0.3756 | -0.198-0.075 | -0.001 | 0.015 | 0.9288 | -0.032-0.029 |  |  |  |  |
|  | 3-month | 0.013 | 0.015 | 0.359 | -0.015-0.042 | 0.034 | 0.045 | 0.4487 | -0.054-0.121 | -0.013 | 0.010 | 0.1815 | -0.032-0.006 |  |  |  |  |
|  | 1-month | 0.020 | 0.014 | 0.1464 | -0.007-0.047 | -0.004 | 0.042 | 0.9195 | -0.086-0.078 | -0.006 | 0.009 | 0.4874 | -0.024-0.012 |  |  |  |  |
| PM0.1 | 12-month | -0.033 | 0.011 | 0.0034 | -0.056--0.011 | -0.003 | 0.034 | 0.9225 | -0.071-0.064 | -0.021 | 0.008 | 0.0073 | -0.036--0.006 |  |  |  |  |
|  | 3-month | -0.010 | 0.011 | 0.3616 | -0.032-0.012 | -0.044 | 0.034 | 0.1901 | -0.11-0.022 | -0.019 | 0.007 | 0.01 | -0.033--0.004 |  |  |  |  |
|  | 1-month | -0.014 | 0.010 | 0.1432 | -0.033-0.005 | -0.012 | 0.030 | 0.6959 | -0.07-0.046 | -0.018 | 0.006 | 0.005 | -0.031--0.005 |  |  |  |  |
| PM2.5 | 12-month | -0.030 | 0.020 | 0.1369 | -0.07-0.01 | 0.055 | 0.061 | 0.3641 | -0.064-0.175 | -0.005 | 0.014 | 0.7259 | -0.031-0.022 |  |  |  |  |
|  | 3-month | -0.009 | 0.011 | 0.3765 | -0.03-0.011 | -0.019 | 0.032 | 0.5563 | -0.082-0.044 | 0.003 | 0.007 | 0.7181 | -0.011-0.016 |  |  |  |  |
|  | 1-month | -0.019 | 0.010 | 0.0565 | -0.038-0.001 | 0.009 | 0.030 | 0.7501 | -0.049-0.068 | -0.005 | 0.007 | 0.4505 | -0.018-0.008 |  |  |  |  |
| PM10 | 12-month | -0.009 | 0.015 | 0.5753 | -0.039-0.022 | 0.003 | 0.047 | 0.9534 | -0.089-0.094 | 0.023 | 0.010 | 0.0293 | 0.002-0.043 |  |  |  |  |
|  | 3-month | -0.006 | 0.008 | 0.4648 | -0.023-0.01 | -0.002 | 0.026 | 0.9331 | -0.052-0.048 | 0.008 | 0.006 | 0.1658 | -0.003-0.019 |  |  |  |  |
|  | 1-month | -0.010 | 0.009 | 0.2448 | -0.027-0.007 | 0.015 | 0.026 | 0.5484 | -0.035-0.066 | 0.009 | 0.006 | 0.1127 | -0.002-0.02 |  |  |  |  |
| Est. estimate; SE. standard error; P. p value; 95% CI. 95% confidence interval. | | | | | | | | | | | | | |  |  |  |  |
